# Broad cross-reactive IgG responses elicited by adjuvanted vaccination with recombinant influenza hemagglutinin (rHA) in ferrets and mice

**DOI:** 10.1371/journal.pone.0193680

**Published:** 2018-04-11

**Authors:** Jiong Wang, Shannon P. Hilchey, Marta DeDiego, Sheldon Perry, Ollivier Hyrien, Aitor Nogales, Jessica Garigen, Fatima Amanat, Nelson Huertas, Florian Krammer, Luis Martinez-Sobrido, David J. Topham, John J. Treanor, Mark Y. Sangster, Martin S. Zand

**Affiliations:** 1 Division of Nephrology, Department of Medicine, University of Rochester Medical Center, Rochester, New York, United States of America; 2 Department of Microbiology and Immunology, University of Rochester Medical Center, Rochester, New York, United States of America; 3 Biostatistics, Bioinformatics, and Epidemiology Program, Vaccine and Infectious Disease Division, Fred Hutchinson Cancer Research Center, Seattle, Washington, United States of America; 4 Division of Allergy, Immunology and Rheumatology, University of Rochester Medical Center, Rochester, New York, United States of America; 5 Department of Microbiology, Icahn School of Medicine at Mount Sinai, New York, New York, United States of America; 6 Division of Infectious Disease, University of Rochester Medical Center, Rochester, New York, United States of America; 7 Rochester Center for Health Informatics, University of Rochester Medical Center, Rochester, New York, United States of America; University of South Dakota, UNITED STATES

## Abstract

Annual immunization against influenza virus is a large international public health effort. Accumulating evidence suggests that antibody mediated cross-reactive immunity against influenza hemagglutinin (HA) strongly correlates with long-lasting cross-protection against influenza virus strains that differ from the primary infection or vaccination strain. However, the optimal strategies for achieving highly cross-reactive antibodies to the influenza virus HA have not yet to be defined. In the current study, using Luminex-based mPlex-Flu assay, developed by our laboratory, to quantitatively measure influenza specific IgG antibody mediated cross-reactivity, we found that prime-boost-boost vaccination of ferrets with rHA proteins admixed with adjuvant elicited higher magnitude and broader cross-reactive antibody responses than that induced by actual influenza viral infection, and this cross-reactive response likely correlated with increased anti-stalk reactive antibodies. We observed a similar phenomenon in mice receiving three sequential vaccinations with rHA proteins from either A/California/07/2009 (H1N1) or A/Hong Kong/1/1968 (H3N2) viruses admixed with Addavax, an MF59-like adjuvant. Using this same mouse vaccination model, we determined that Addavax plays a more significant role in the initial priming event than in subsequent boosts. We also characterized the generation of cross-reactive antibody secreting cells (ASCs) and memory B cells (MBCs) when comparing vaccination to viral infection. We have also found that adjuvant plays a critical role in the generation of long-lived ASCs and MBCs cross-reactive to influenza viruses as a result of vaccination with rHA of influenza virus, and the observed increase in stalk-reactive antibodies likely contributes to this IgG mediated broad cross-reactivity.

## Introduction

Seasonal influenza virus infection is one of the largest recurring global public health threats [1]. Antibody mediated protection against infection by influenza A and B viruses arises from prior infection and vaccination. Infection induces adaptive immunity mediated by both antibodies and virus reactive T cells, while vaccination primarily elicits a T cell dependent IgG response directed against influenza virus hemagglutinin (HA) and neuraminidase (NA) proteins[2, 3]. HA is responsible for viral binding to host cell surface sialic acid residues and for viral fusion. Most neutralizing anti-influenza virus antibodies target the HA protein head region, which contains the sialic acid receptor-binding site. Antibodies targeting this region can neutralize the virus by blocking binding to sialic acid residues on the surface of target cells [4].

Eighteen subtypes of influenza virus HA are currently known and are clustered into two phylogenic groups based on sequence similarity and serological cross-reactivity: influenza group 1 (H1, H2, H5, H6, H8, H9, H11, H12, H13, H16, H17 and H18) and group 2 (H3, H4, H7, H10, H14 and H15) [4]. Most anti-influenza IgG is directed against the globular head of the HA, and is specific to viral strains and subtypes [5]. High viral HA mutation rates provide a mechanism for influenza viruses to evade pre-existing anti-HA globular head IgG Abs. For this reason, influenza virus vaccines are reformulated annually based on viral surveillance data to match the prevalent circulating strains[6]. Even so, variability in circulating viruses and poor responses to vaccination decrease vaccine efficacy [7].

In contrast to the head domain, the stalk domain of the HA protein is relatively conserved, and antibodies targeting this region generally cross-react with many viral subtypes within the same phylogenic group [8]. Anti-stalk antibodies also protect against infection by preventing viral envelope-cell membrane fusion [9, 10], and facilitating Fc-mediated antibody dependent cellular cytotoxicity (ADCC) and complement dependent lysis (CDL) [11–13]. There is, however, limited clinical evidence regarding the relative efficacy of antibodies directed against the HA stalk domain as compared to those targeting the HA head [14]. Thus, a major objective of current influenza vaccination approaches is to devise strategies that increase such broadly reactive anti-influenza virus IgG antibodies.

Development of broadly cross-reactive anti-influenza vaccine responses is challenging given the heterogeneity of influenza viruses, especially with respect to HA protein. Recently, several studies have suggested that antibody mediated cross-reactive immunity against the influenza virus HA protein strongly correlates with long-lasting cross-protection against influenza strains that differ from the primary infection or vaccination strain [15–17]. Induction of significant amounts of broadly cross-reactive and protective antibodies against influenza viruses is an important goal for vaccine design, as current vaccination strategies do not induce high titers of cross-reactive protective antibodies. One recent approach has been to target the conserved stalk domain of HA. Another strategy, designed to break the immunodominance of the head domain, is sequential vaccination with chimeric HAs (cHAs) that have different head domains but the same stalk domain [18–21]. Serial vaccination, in combination with adjuvant, with such recombinant cHAs induced broad cross-reactive humoral immunity, resulting in protection from heterosubtypic influenza viral challenge [19].

An alternative approach to inducing broad cross-reactive immunity to multiple influenza virus strains is adjuvanted intramuscular vaccination [22]. For example, MF59, a squalene-based oil-in-water emulsion, has been clinically approved for use with influenza vaccination in adults >65 years of age, who typically respond poorly to traditional influenza vaccines. Compared with unadjuvanted intramuscular influenza vaccination, the addition of MF59 significantly improves long-lasting, vaccine strain specific, CD8 T cell and anti-HA IgG responses [23]. Importantly, it has been reported that the addition of MF59 increases broad antibody cross-binding to influenza strains not present in the original vaccine [24, 25]. Addavax, a squalene-based oil-in-water nano-emulsion with a formulation similar to that of the clinical formulation of MF59, but for research use, has also shown equivalent effects [21].

These promising observations regarding induction of IgG mediated cross-reactive immunity raise several key issues and questions. For example, while cross-reactive immunity can be broadly described, no method exists for quantifying its intensity and breadth. Also, how the vaccination schedule affects any resulting cross-reactive immunity is poorly understood. Finally, the role and timing of adjuvant within the vaccine schedule also remains unclear. To address these questions, we quantified cross-reactive antibodies induced by prime-boost-boost adjuvanted rHAs in ferrets and mice by measuring reactivity to 29 influenza HAs using our recently described mPlex-Flu assay [26], combined with an assessment of vaccine-induced immune repertoire cartography and stalk-reactive antibodies. The results demonstrated the critical role of vaccine schedule timing of adjuvant inclusion in a sequential vaccination strategy, and show that primary vaccination with Addavax adjuvanted rHA could be a key strategy to induce broadly cross-reactive and protective anti-influenza HA IgG responses.

## Material and methods

### Animal and ethics statement

For all mouse experiments, we used naïve female 9-10-week-old C57BL/6 mice obtained from Taconic Biosciences (Rensselaer, NY). All experiments in mice were conducted according to protocols approved by the University Committee on Animal Resources (UCAR) of University of Rochester (protocol number #2011–055). Four to five mice were housed inside ventilated cabinet under controlled temperature with daily monitoring in a BSL-2 pathogen–free condition animal facility. Mice were routinely observed throughout experiments for any signs of distress, clinical symptoms of illness by trained personnel. Mice were euthanized also according to the UCAR protocol.

### Recombinant HA proteins

All rHAs of type A influenza viruses in this study were expressed using a recombinant baculovirus system based on a modified pFastBac vector with a C-terminal trimerization domain and a hexahistidine purification tag [27]. We also subcloned all HA genes coding B type influenza viruses into this pFastBac vector using *BamHI* and *NotI* restriction endonucleases (NEB, Ipswich, MA). Briefly, we designed primer pairs containing *BamHI* and *NotI* restriction enzyme sites to amplify the first 521 aa of the HAs, and subcloned these HA fragments into the baculovirus shuttle vector. Primer sequences are available upon request.

Expression and purification of rHA was performed as previously described [27]. Purified rHAs were concentrated and desalted with 30 kDa Amicon Ultracell centrifugation units (Millipore, Billerica, MA) and re-suspended in phosphate buffered saline (PBS, pH7.4). The purity, integrity and identity of proteins were assessed by NuPage 4–12% Bis-Tris gels (Invitrogen, Grand Island, NY) and Western blot (S1 Fig). Western blot analysis was performed using rabbit anti-influenza strain/subtype specific polyclonal primary (eEnzyme, Gaithersburg, MD) and goat anti-rabbit horseradish peroxidase conjugated secondary (Bio-Rad, Hercules, CA) antibodies. Protein concentration was quantified using the Quickstart Bradford Dye Reagent (Bio-Rad, Hercules, CA) with a bovine serum albumin standard curve.

### Adjuvant and specific anti-sera against influenza viruses from ferrets and mice

Anti-influenza ferret antisera (Table 1) were obtained from the Influenza Research Resource (IRR, Manassas, VA). They were drawn after either primary infection with influenza virus, or after three consecutive subcutaneous vaccinations with non-hexahistidine tagged rHA proteins admixed with complete Freund’s adjuvant for prime, and boosted twice with incomplete Freund’s adjuvant. Per the manufacturer, sera were collected after the third vaccination at peak response after the third vaccination and pooled. We independently validated and determined sera dilutions for the mPlex-Flu assay by assaying the ferret sera against the vaccine-homologous rHA. For experiments with ferret sera, each serum sample was diluted to obtain a similar mPlex-Flu assay MFI level (~15,000) against the vaccine-homologous HA for that sera. These generally were close to the HAI or ELISA titers provided by the manufacturer, summarized in Table 1.

**Table 1:** Ferret polyclonal anti-HA serum from the Influenza Research Resource (IRR).

	Catalog No	Induce antigen or virus	Manufacturer HAI titer*	Dilution
**H1N1**	FR-359	A/California/07/2009 (H1N1) pdm09	5120	5120
	FR-291	rHA A/California/04/2009 (H1N1) pdm09**	N/D	6000
	FR-388	A/Brisbane/59/2007	1280	1280
	FR-288	rHA A/Brisbane/59/2007**	N/D	6000
	FR-390	A/Mexico/4108/2009 (H1N1)	5120	5000
	FR-393	A/Utah/20/2009 (H1N1)	2560	2500
	FR-953	A/USSR/90/1977 (H1N1)	640	500
**H3N2**	FR-289	rHA A/Brisbane/10/2007 (H3N2)**	N/D	5000
	FR-292	rHA A/Hiroshima/52/2005 H3N2**	N/D	5000
	FR-389	A/Brisbane/10/2007 (H3N2)**	5120	5000
	FR-445	A/Wisconsin/15/2009 H3N2	2560	2500
	FR-446	A/Perth/16/2009 (H3N2)	5120	5000
	FR-646	A/Victoria/210/2009 (H3N2)	5120	5000
	FR-950	A/Indiana/10/2011 (H3N2)	640	500
	FR-1079	A/Victoria/361/2011 (H3N2)	320	250
**H5N1**	FR-960	A/Vietnam/1203/2004(H5N1	1280	1200
	FR-708	rHA A/Vietnam/1203/2004 (H5N1)**	N/D	6000
	FR-782	A/Anhui/01/2005 (H5N1) PR8-IBCDC-RG6	2560	2500
	FR-1083	A/India/NIV/2006 (H5N1) PR8-IBCDC-RG7	320	500
	FR-1086	A/Hubei/1/2010 (H5N1) PR8-IDCDC-RG30	1280	1200

* IRR provided titer from manufacturer performed HAI titration

** non-His-tagged full length rHA protein vaccinated; (ELISA titers were performed by IRR)

N/D = no HAI assay was performed by IRR

For mouse experiments, we generated anti-influenza anti-sera by vaccinating female C57BL6 mice with recombinant influenza HA protein with or without Addavax (InvivoGen INC, San Diego, CA), a squalene-based oil-in-water nano-emusion with a formulation similar to MF59, per the manufacturer’s instructions.

### mPlex-Flu assay

Purified rHA proteins for each influenza virus strain/subtype (Table 2) were covalently coupled to Bio-plex Pro^™^ Magnetic COOH Beads (Bio-Rad, Hercules, CA) using the Bio-Plex Amine Coupling Kit (Bio-Rad, Hercules, CA) according to the manufacturer’s instructions, as previously described [26]. The coupled beads were tested with anti-HA subtype specific ferret or rabbit polyclonal antibodies (Influenza Reagent Resource, IRR, Manassas, VA), to confirm antigen binding and density. Secondary antibodies were polyclonal goat anti-rabbit (catalogue #4010–09, raised against rabbit IgG; dilution 1:400, SouthernBiotech, AL) and polyclonal goat-anti-ferret antibodies (catalogue sab4024, raised against ferret IgG; dilution 1:400, Brookwood Biomedical, Birmingham, AL), respectively.

**Table 2:** The HA panel of the mPlex-Flu assay.

Category	Flu strain	Abbreviation	Genebank Accession No
H1	A/South Carolina/01/1918	A/SC18	AF117241.1
	A/PR/8/1934	A/PR8	CY148243.1
	A/USSR/1977	A/USSR77	DQ508897.1
	**A/Texas/36/1991**	**A/Tex91**	DQ508889.1
	**A/New Caledonia/20/1999**	**A/NewCal99**	CY125100.1
	**A/California/07/2009**^#^	**A/Cal09**	FJ966974.1
H2	A/Japan/305/1957	A/Jap57	L20407.1
H3	A/Hong Kong/1/1968^#^	A/HK68	CY112249.1
	A/Port Chalmers/1/1973^#^	A/PC73	CY009348.1
	**A/Perth/16/2009**	**A/Per09**	GQ293081.1
	**A/Victoria/361/2011**	**A/Vic11**	KM821347
	**A/Texas /50/2012**^#^	**A/Tex12**	KC892248.1
H5	A/Vietnam/1204/2004	A/Vie04	EF541403.1
	A/Indonesia/05/2005	A/Ind05	EF541394.1
H6	A/Taiwan/2/2013	A/TW13	KJ162860.1
H7	A/rhea/North Carolina/39482/1993	A/NC/93	EF470586
	A/mallard/Netherlands/12/2000	A/Net00	KF695239
H9	A/guinea fowl/HongKong/WF10/1999	A/gfHK99	AY206676.1
Modified HA	H5 head (A/Vie04)	H5 head	
	H7 head (A/ShangHai/1/2013, A/SH13)	H7 head	
	H9 head (A/gfHK99)	H9 head	
Chimeric HA	cH5/1PR8 (A/Vie04, A/PR8)	cH5/1PR	
	cH5/1Cal09(A/Vie04, A/Cal09)	cH5/1Cal	
	cH4/7 (A/duck/Czech/1956, A/SH13)	cH4/7	
	cH9/1Cal09(A/gfHK99, A/Cal09)	cH9/1Cal	

Seasonal Vaccine strains in Bold

^#^ Subcloned by our laboratory.

Quantitation of IgG levels in anti-sera of ferrets and mice was performed as previously described [26]. All mPlex-Flu assays for each experiment were performed at the same time to minimize batch effects. Briefly, a panel of HA-coupled mPlex-Flu beads (Table 2) were mixed and incubated with diluted ferret or mouse serum, at 500 beads per each HA to be detected, in the 96-well filtration plates (Millipore, Billerica, MA) at 4°C overnight, on a rotary shaker (500 rpm), in the dark. Wells were washed twice and then incubated with 1:400 diluted PE conjugated anti-mouse IgG (γ chain specific) secondary antibodies (catalogue #1030–09, SouthernBiotech, AL) in the dark at room temperature for 2 hours with gentle agitation (500 RPM). After three additional washes, the beads in each well were suspended in Luminex Magpix Drive Fluid (Luminex, Austin, TX), analyzed on a MagPix multiplex reader (Luminex, Austin, TX), and the results expressed as median fluorescence intensity (MFI). Absolute concentrations of each anti-HA IgG present in serum were calculated from a standard curve generated at same time with a mixture of murine positive anti-sera as previously described[26].

For control experiments, a 46 amino acid peptide (TT6H, >98% purity) containing a thrombin cleavage site, a trimerization domain from bacteriophage T4 fibritin protein (Fd), and a histidine (His) tag (sharing the same protein sequence as the C-terminal of rHAs) was synthesized by Genscript (Piscataway, NJ). The TT6H peptide was coupled to Luminex beads using the Bio-Plex Amine Coupling Kit (Bio-Rad, Hercules, CA) at the same molar concentration used to couple the rHAs. The TT6H coupled beads were mixed with other rHA coupled beads to test binding of His-tagged specific antibodies using a monoclonal anti-C terminal His-tag antibody (R930-25, Invitrogen INC.Carlsbad, CA). Fd specific antibodies were assessed using rabbit polyclonal anti-trimerization Fd domain antisera provided by Dr. Sanders (Weill Medical College of Cornell University, NY), generated by vaccinating rabbits three times with Fd domain fused to HIV envelope (YU2 gp140) protein without His-tag (Env-Fd) [28].

### *In vivo* immunization experiments

**Fig 1:**
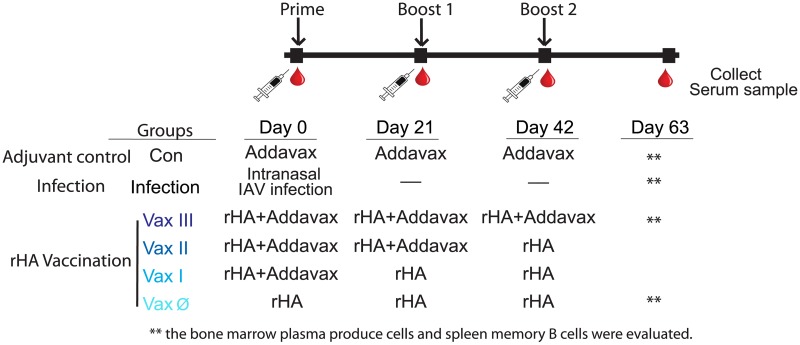
Vaccination strategy and schedule. Two cohorts of mice were vaccinated with A/California/07/2009 (A/Cal09, H1N1) and A/Hong Kong/1/1968 (A/HK68, H3N2) influenza virus rHA, respectively. A negative control group was included, consisting of vaccination with Addavax alone; the infection group consisted of intranasal infection with of same influenza viral strains at day 0 for both cohorts. rHA vaccinations (Vax) were delivered three weeks apart, with or without Addavax adjuvant as indicated. Individual mice were bled at pre-vaccination (day 0), and at 21, 42, and 63 days post vaccination, and sacrificed at day 63 for collection of spleen and bone marrow cells.

*In vivo mouse* vaccination study, we used Addavax as adjuvant. and vaccinated intramuscularly (IM) with rHA either from A/Hong Kong/1/1968 (A/HK68) or A/California/07/2009 (A/Cal09) viruses, with or without 10μg Addavax mixed with rHA protein at 1:1 ratio in PBS, followed by boosting (with or without adjuvant) at 21 and 42 days after the primary injection (Fig 1). An adjuvant-only control group received intramuscular injection with Addavax alone. In those two cohorts, while mice in the infection groups received an intranasal inoculation with either A/HK68 virus at 10^5^ ECID_50_ or mouse adapted A/Cal09 virus at 200 ECID_50_. Animals from all groups (n = 6) underwent retro-orbital phlebotomy at baseline (day 0) and on days 21, 42 and 63-days post-vaccination. Animals in adjuvant control, infection and rHA vaccine three time with adjuvant group (Vax III) were euthanized on day 63 to harvest spleen and bone marrow cells (Fig 1) for ASC and memory B cell assays.

### ELISpot assay for detection of cross-reactive IgG antibody secreting cells (ASCs)

Vaccine-specific IgG ASCs were estimated by ELISpot assay as previously described [29]. Briefly, 96-well ELISpot plates (EMD Millipore, Billerica, MA) were coated for one hour at room temperature with 50 μl of 8 μg/mL goat-anti-mouse IgG (KPL, Milford, MA) for assessing total IgG, or 50 μL of a solution containing purified HA protein from the B/Bris08 strain (20 μg/mL) as the background control (to subtract any potential positive B cell responses directed against the thrombin cleavage site, the trimerization domain (Fd) and/or the His-tag region), or 50 μL containing 20 μg/mL purified rHA proteins from the A/Cal09 and A/HK68 strains. Wells were washed with phosphate buffered saline (PBS) and then blocked for one hour at 37°C with 200 μL/well of Roswell Park Memorial Institute medium (RPMI; Gibco, Waltham, MA) culture media containing 10% heat inactivated fetal bovine serum (FBS; Gibco, Waltham, MA). Single marrow cells were extracted from both femurs from each mouse with Iscove’s Modified Dulbecco’s Medium (IMDM; Gibco, Waltham, MA). Cells were then washed twice with PBS containing 0.5% Bovine Serum Albumin (BSA), and added to the pre-coated plates at 500,000 cells/well and 1:2 serial dilutions performed to 6,250 cells/well. The plates were incubated for four hours in a 5% CO_2_ humidified incubator at 37°C. After the incubation, the wells were washed six times with 0.1% Tween-20 PBS (PBST, Sigma-Aldrich, St. Louis, MO) and phosphatase-labeled goat anti-mouse IgG detection antibody (catalog #1030–50, SouthernBiotech, AL) added at 100 μL/well. Plates were then incubated overnight at 4°C, washed six times with PBST, then IgG specific spots were developed using the Alkaline Phosphate Substrate Kit III (Vector Laboratories, Burlingame, Ca). Spots numbers were enumerated using an ImmunoSpot plate reader (Cellular Technology Limited, Cleveland, OH).

### HA specific memory B cells (MBCs)

Single cell suspensions were prepared from spleen in complete IMDM medium (Gibco, Waltham, MA) with 10% FBS, 100 units/mL of penicillin, 100 μg/mL of streptomycin, and 5×10^−5^ M of 2-mercaptoethanol (Sigma-Aldrich, St. Louis, MO). Lymphocytes were counted and diluted to 5×10^6^ cells/mL. 2.5×10^6^ cells/well were added into 48-well plates. For lipopolysaccharides (LPS) stimulation wells, cells were cultured with 0.4 μg R595 LPS (Sigma-Aldrich, St. Louis, MO), and 100 μL of supernatant from concanavalin A-stimulated splenocytes, prepared as previously described [30]. Influenza virus-specific *in vitro* stimulation was performed as previously described [31]. Briefly, mouse splenocytes were cultured for 6 days with 10 μg β-propiolactone (BPL)-inactivated A/Cal09 (IRR, FR1184), H3N2 control antigen 2009–2013 (FR43), and B/Brisbane/08 (B/Bris08, FR 1188), respectively. The culture supernatants were harvested and stored at -80°C for mPlex-Flu assay. For the mPlex-Flu assay, all samples were thawed, diluted and assayed at same time to minimize batch effects.

### Viral neutralization by post-infection and post-vaccination mouse sera

A/HK68, A/Cal09, A/Puerto Rico/8/1934 (A/PR8) and B/Bris08 influenza viruses were provide by Dr. Martinez-Sobrido (University of Rochester, NY). A/Victoria/361/2011 (A/Vic11, H3N2) influenza virus was obtained from the Influenza Research Resource (IRR, Manassas, VA). Viruses were titrated as previously described [32]. Briefly, confluent plates of Madin-Darby canine kidney (MDCK, ATCC CCL-34) cells (96-well format, 5x10^4^ cells/well) were infected with 10-fold serial dilutions of tissue culture supernatants. At 8 hours post infection, cells were fixed and permeabilized using 4% formaldehyde, 0.5% triton-X100 in PBS for 30 minutes at room temperature. Cells were then washed thrice with PBS and incubated in PBS/2.5% bovine serum albumin (BSA) for one hour at room temperature. Cells were washed three more times with PBS, and incubated with the influenza A virus nucleoprotein (NP) monoclonal antibody HB-65 (ATCC, H16-L10-4R5), diluted in 1% BSA for two hours at 37°C. After washing three more times with PBS, cells were incubated with a fluorescein isothiocyanate (FITC)-conjugated rabbit anti-mouse IgG secondary antibody (Dako) diluted 1:1,000 in 1% BSA in PBS, for one hour at 37°C. IAV NP-positive cells were visualized and enumerated to determine virus titers (fluorescent forming units, FFU/mL) using a fluorescence microscope.

For microneutralization (MN) assays [33], two-fold serial dilutions of heat-inactivated mouse sera were mixed with approximately 50 FFU of each virus and was left at room temperature for 1 hour. Confluent MDCK cell monolayers were then inoculated with the serum/virus mixtures. After an absorption period of 60 minutes at room temperature, serum/virus mixtures were removed and replaced with DMEM (Gibco, Waltham, MA) supplemented with 0.3% BSA, 10 μg/mL gentamycin, and 1 μg/mL TPCK-trypsin in 96-well plates for 4-repeat wells that were incubated for a further 4 days, and the cytopathic effect was examined by crystal violet staining. Neutralization titers were defined as the last dilution at which infection was completely blocked in 50% of the wells, and geometric mean titers (GMT) were calculated from 3 independent assays.

### Data analysis and visualization

Antigenic dissimilarities between rHA strains were visualized by projecting 28-dimensional HA protein sequences onto two-dimensional subspaces using multi-dimensional scaling [26, 34]. To compare antigenic properties of rHA strains, we used a functional feature vector approach [35]. For each HA amino acid sequence, we calculated a 60-component normalized feature vector that measures protein relatedness based on the distribution of amino acids in the HA protein sequences, including position and protein charge. This approach has the advantage of being independent of variations in protein length, while accounting for sequence differences as well as physical properties of the proteins that affect antigenicity (e.g. charge, hydrophobicity) [35]. An antigenic dendrogram was then generated by hierarchical clustering using the squared Euclidean distance. Cluster fusion levels were determined based on Unweighted Pair Group Method with Arithmetic Mean (UPGMA) linkage criteria [34]. All analyses were performed using *Mathematica* (version 10, Wolfram Research).

The study design included six experimental groups (an adjuvant alone control, infected, and four vaccination groups: Vax III, Vax II, Vax I and Vax Ø) (Fig 1). Six C57BL/6 female mice were randomized selected to each experimental group. In each mouse, measurements were longitudinally collected at four time points (baseline, and days 21, 42 and 63 post-infection). Relative IgG concentrations (either total or virus HA strain-specific IgG) were estimated based on MFI obtained from serially diluted sera via inverse regression [36]. The primary goal of the analysis was to compare relative concentrations between experimental groups. To reduce skewness in the data, the estimated concentrations were log transformed such that x’ = log(x+1). No differences were expected between experimental groups at baseline, and the corresponding observations were excluded from the analysis to simplify statistical models. The analysis was conducted using linear mixed models [37] including experimental group and time as categorical predictors. We also considered the inclusion of random slopes for time and/or intercepts to capture potential mouse-to-mouse variation. In these analyses, the variable time was treated as continuous in the random effects part of the model. All analyses were carried out using SAS/STAT software, version 9.3 (SAS Institute Inc). All tests were two-sided. P-values were adjusted in post-hoc analyses for multiple comparisons using Tukey’s method. Statistical significance for ELISpot data and neutralization titers were calculated using grouped multiple *t*-tests for multiple comparisons with Prism v7.0 (Graphpad Software).

Complete data and code used to generate figures and analyses are available on figshare.com at https://doi.org/10.6084/m9.figshare.c.4026268.v1.

## Results

### Sequential adjuvanted vaccination with rHA elicits broadly cross-reactive anti-HA IgG

**Fig 2:**
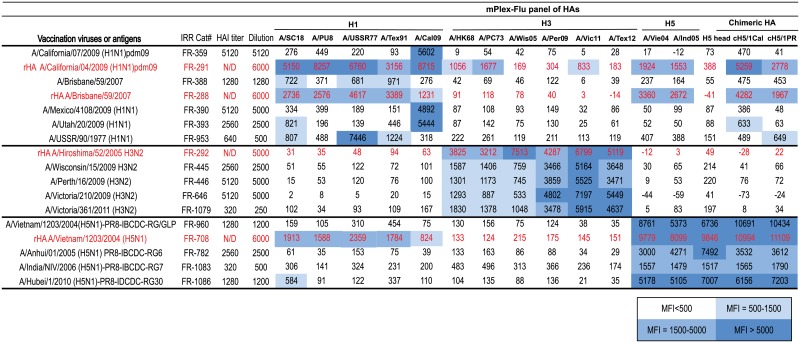
Freund’s adjuvanted rHA protein vaccination elicits broader antibody mediated cross-reactivity compared to viral infection in ferrets. The antibody response against homologuous and cross-reactive HA proteins from different influenza virus strains were evaluated by the mPlex-Flu assay. Samples included ferret reference antisera obtained from the Influenza Research Resource collected after viral infection (black) or after a prime-boost-boost vaccination protocol (see Methods) with Freund’s adjuvant admixed rHA (red). Dilutions of polyclonal antisera are provided in Table 1. The results calculated by subtracting the blank from mean MFI for each influenza virus strain with three replicate wells per influenza strain.

We first compared the breadth and depth of cross-reactive IgG antibodies induced after infection with influenza virus versus vaccination three times (prime-boost-boost) with Freund’s adjuvanted non-His-tagged rHA proteins using ferret reference serum (Table 1). The mPlex-Flu assay allowed us to test both within strain HA head reactivity, cross-strain binding, and cross-phylogenic group binding[26]. We tested post-vaccination and post-infection ferret sera against a panel of 16 rHAs that included major H1(group 1) and H3 (group 2) circulating or vaccine influenza strain subtypes. Details of those purified rHAs (S1 Fig) and control experiments with strain specific monoclonal antibodies are provided in S2 Fig. The results (Fig 2) showed that sera collected from 9 different H1 or H5 influenza virus infections had very specific antibody reactions against homologous or similar strains within the same subtype, but minimal cross-reactivity against other influenza rHAs within the same phylogenic group. In contrast, we found that the antisera generated by vaccination with rHAs from H1N1 strains A/California/04/2009 (A/Cal09) or A/Brisbane/10/2007 (A/Bris08) showed broader cross-reactivity within the H1 subtype, and to H5N1 influenza strains of the same phylogenetic subtype (in red on Fig 2). Interestingly, sera from ferrets vaccinated with adjuvanted A/Cal09 rHA protein (group 1 influenza), also showed low level binding against the A/HongKong/1/1968 (A/HK68) and A/Port Chalmers/01/1973 (A/PC73) H3 strains, which express antigenically more distant phylogenic group 2 HAs (Fig 2).

Multiple vaccination with H1 and H5 rHAs in combination with Freund’s adjuvant produced ferret sera IgG that reacted against both H1 and H5 rHAs (Fig 2). Similarly, sera from ferrets multiply vaccinated with rHA from A/Vietnam/1203/2004 (A/Vie04, H5N1) virus also showed robust cross-reactivity to both H5 and H1 rHAs. In contrast, multiple vaccination of ferrets with H3 influenza rHA, or infection with H3N2 viruses, induced a similar pattern of cross-reactive antibodies within the H3 subtype to all strains tested. Although the mPlex-Flu assay panel in these experiments did not include an rHA protein to test A/Hiroshima/52/2005 (A/Hir05) specific binding, sera from ferrets vaccinated with A/Hir05 rHA produced a stronger cross-reaction to historic A/HK68 and A/PC73 H3 strains than produced by infection with A/Wisconsin/15/2009 (A/Wis09), A/Perth/16/2009 (A/Per09), A/Victoria/210/2009 (A/Vic09) and A/Victoria/361/2011 (A/Vic11) strains (P<0.001).

**Fig 3:**
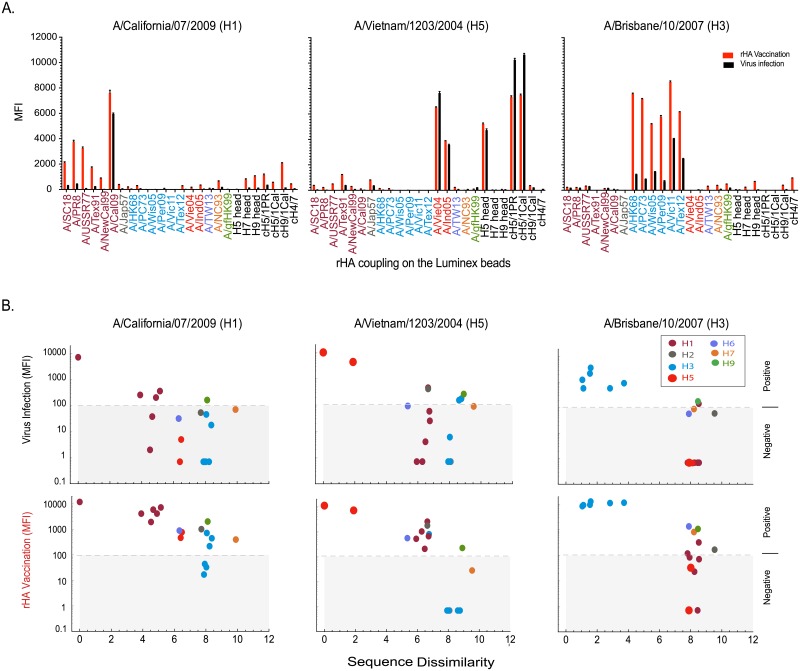
Comparison of antibody mediated cross-reactive immunity induced by influenza infection versus adjuvanted rHA vaccination in ferrets. Post-vaccination serum from ferrets infected with A/California/07/2009 (A/Cal, H1N1), A/Vietnam/1203/2004 (A/Vie04, H5N1) and A/Brisbane/10/2007 (A/Bris07, H3N2) or multiply vaccinated with rHA with Freund’s adjuvant in a prime-boost-boost series were analyzed for IgG reactivity against 25 influenza rHA by multiplex assay. **(A)** Comparison of IgG antibody responses of vaccination versus infection. The results are expressed as the average MFI subtracting blank MFI for each strain (n = 3). The strains are colored according to the different groups. **(B)** A cross-reactivity plot of the IgG binding data for each influenza strain versus the protein sequence-feature dissimilarity of vaccine-homologous HA and each of the vaccine-heterologous rHA proteins used in mPlex-Flu assay. The protein sequence dissimilarities of rHAs were calculated using a protein feature vector approach[34, 35] and Euclidean distance (S3 Fig). The positive cut-off MFI unit is 100 (based on the average of negative control sera).

The above findings suggested that adjuvanted rHA multiple vaccinations might produce much broader influenza strain cross reactivity, and thus we next tested the reactivity of ferret antisera IgG generated by either multiple (prime-boost-boost) rHA vaccinations or primary infection with the A/Cal09(H1N1), A/Vie04(H5N1) and A/Brisbane/10/2007 (A/Bris07, H3N2) viruses. In these experiments, serum reactivity was measured using an expanded 29-strain mPlex-Flu assay panel with a broad array of influenza virus rHAs, including both phylogenetic group 1 (H1, H2, H5, H6, H9) and group 2 (H3, H4, H7) strain. To better assess anti-HA head versus stalk reactivity, the panel also included several chimeric rHAs (cHAs) consisting of the head domain from one viral subtype and the stalk from a different subtype, including cH5/1_Cal09_, cH5/1_PR8_, and cH9/1_Cal09_ (for group 1 stalk-reactive IgG detection) and cH4/7_A/SH13_ (for group 2 testing). The results (Fig 3A) confirmed that vaccination with the rHAs group 1 HA from the A/Cal09 virus induced significant IgG cross-reactivity to strains within the same H1 (covering 90 years of antigenic variation) or H5 subtypes, and broader cross-reactivity to other influenza virus HAs within the same phylogenic group (H2, and H5). These experiments also confirmed that the vaccine-induced anti-sera against A/Bris07 (H3) showed significantly broader IgG cross-reaction to rHAs within the H3 subtype panel (covering 50 years of antigenic variation) and weak reaction to H7 rHAs within the same phylogenic group. Additionally, a low level of stalk-reactive IgG antibodies were detected in both the H1 and H3 rHA vaccination groups, which were significantly higher than those elicited by infection with the identical viral strain (P<0.001).

To directly visualize the relationship of the cross-reactive responses induced by vaccination/infection to the antigenic similarity of the viruses, we also calculated the protein sequence dissimilarity matrix of the influenza rHA panel using a functional feature vector approach (S3 Fig) [34, 35]. We then plotted the degree of rHA dissimilarity against the mPlex-Flu IgG reactivity measurements after vaccination or infection (Fig 3B). This allowed us to visualize the degree to which post-exposure (infection or rHA vaccination) anti-HA IgG reactivity for each rHA strain was influenced by the estimated rHA antigenic similarity to the challenge strain (Fig 3B). The results of A/Cal09 H1 group indicated that the cross-reactive IgG binding patterns between influenza rHA were highly correlated with the antigenic similarity of the rHAs; the greater the similarity between HAs, the greater the induction of cross-reactive antibody responses, as expected. For the A/Bris07 H3 group, vaccination did not significantly change the breadth of cross-reactive antibodies compared to infection.

### Addavax adjuvanted rHA vaccination with multiple inoculations elicits broader cross-reactivity than influenza infection in mice

The ferret sera in the above experiments were generated by rHA and Freund’s adjuvant, and designed to be used as control sera in HAI assays. To more rigorously investigate the potential effect of adjuvant in prime and boost vaccination protocols, we generated anti-HA sera by vaccinating mice with rHA and the adjuvant Addavax [21], a squalene-based oil-in-water emulsion. Addavax, a research use reagent, is similar to MF59, which has been clinically approved for adjuvant use in human flu vaccinatio. We were particularly interested in cross-reactivity induced by vaccination with multiple doses of rHA adjuvanted Addvax in the mouse model given the potential clinical relevance to MF59 vaccine protocols. Several groups have reported that sequential vaccination of mice with chimeric HAs, having the same stalk region but different head region, or infection with different HA head region viruses, could elicit higher levels of HA stalk-reactive antibodies that contribute the cross-protective [19, 38].

Based on the above observations, we hypothesized that Addavax adjuvanted vaccination would also increase and broaden the IgG response to rHA in mice. Experiments were designed to ask four questions: (1) What is the degree of cross-reactivity induced by MF59-like adjuvant rHA vaccination compared with infection? (2) If there is broader cross-reactive binding with MF59-like adjuvanted rHA vaccination, is this functionally protective against viral infection? (3) What is the optimal timing to include MF59-like adjuvanted in a multiple-boost vaccination strategy if the goal is to induce broad cross-reactivity? (4) What is the contribution of stalk-reactive antibodies to any MF59-like adjuvant induced cross-reactivity?

**Fig 4:**
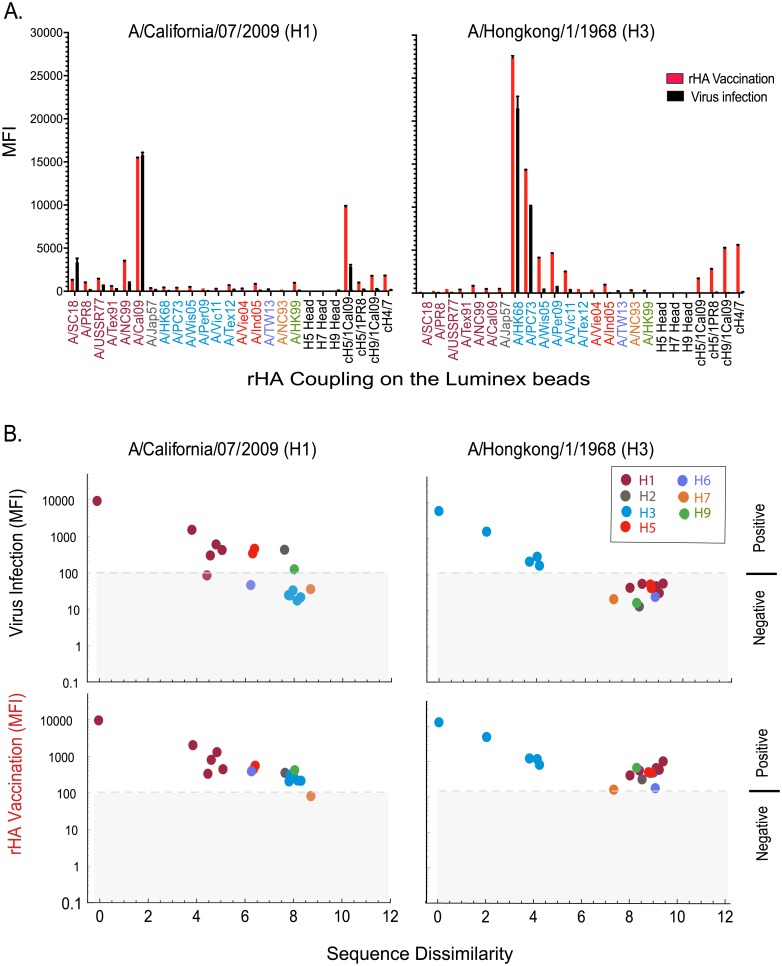
Comparison of cross-reactivity of serum IgG from influenza virus infected versus Addavax adjuvanted rHA vaccinated mice. (**A**) Multiplex assay comparison of IgG seroreactivity against a panel of 29 influenza rHA resulting from viral infection or the prime-boost-boost vaccination with Addavax (MF59-like) admixed rHAs from either A/California/07/2009 (A/Cal09, H1N1) or A/HongKong/1/1968 (A/HK68, H3N2). Sera were collected 9 weeks post- infection, or 3 weeks after the final vaccine boost (day 63 after the priming vaccination). Sera harvested from six mice per group were pooled for analysis. The IgG binding against influenza HA are reported as the mean MFI minus the baseline of each strain (n = 3). (**B**) A cross-reactivity plot of the mouse serum IgG binding data for each influenza virus strain versus the protein feature dissimilarity of vaccine HA and each of the rHA proteins used in mPlex-Flu assay.

To answer these questions, we performed a series of vaccinations with Addavax adjuvanted rHA, A/Cal09 (H1) or A/HK68 (H3), and compared these with homologous rHA vaccination or intranasal infection after 9 weeks (vaccination strategy shown in Fig 1). The results (Fig 4), similar to those observed in ferrets, showed that compared to viral infection, multiple intramuscular vaccinations with Addavax adjuvanted rHA of A/HK68 significantly increased cross-reaction responses against influenza HA within viral subtypes and phylogenic groups. In addition, adjuvanted vaccination appeared to produce higher levels of stalk-reactive antibodies, as suggested by the binding of mouse sera to the chimeric proteins cH5/1, cH9/1 and cH4/7, and the absence of binding to H5 and H9 containing only the head domain of the protein (Fig 4A). These results suggest that multiple adjuvanted rHA vaccination elicited broader cross-reaction within subtype, and higher levels of stalk-reactive antibodies might contribute to this effect.

Unfortunately, post-expression cleavage of the trimerization domain from the A/Cal09 (H1) rHA protein led to degradation of protein under multiple conditions (S4 Fig). Thus, we were not able to generate a stable A/Cal09 (H1) rHA ectodomain without the TT6H tag including trimerization (Fd) and hexahistidine(His) tags for vaccination. Experiments performed with the full protein raised a question whether C-terminal tagged rHA could induce detectable antibodies against those tags [39]. However, mPlex-Flu assay analysis of anti-His mAb and anti-Fd rabbit polyclonal anti-serum against rHA-TTH6 demonstrated very low binding, similar to that observed naïve-mouse or rabbit sera (S5 Fig). These results suggest that the trimerization domain and His-tag on the C-terminal of rHAs, when coupled to the Luminex bread surface, are most likely sterically masked by the large rHA protein and density of proteins on the bead surface, appearing “silent” even in the presence of high concentrations of specific antibodies. Therefore, the binding observed in mPlex-Flu assay, shown above, is due to the binding to the HA protein component, and not to the TT6H tag.

### Priming with Addavax adjuvant and rHA is essential for the induction of broad cross-reactive antibodies

Next, we investigated the optimal timing of admixing Addavax in a multiple-boost vaccination strategy for induction of broad cross-reactive antibodies. It is known that rHA protein is poorly immunogenic, and that the addition of oil-in-water emulsion adjuvants (e.g. MF59, AS03, Addavax, etc.) enhance vaccine antigenicity, but typically require at least 2 doses [21, 25]. These results are generally interpreted to indicate that adjuvant augmented antigen priming induces long-lasting cross-reactive immune memory [40]. In order to better understand the role of oil-in-water emulsion adjuvant in cross-reactive responses to rHA protein vaccination, we performed sequential vaccination experiments in mice that used priming-boosting with or without Addavax. Two control groups were included: (1) mice intranasally infected with either A/HK68 (H3) and A/Cal09 (H1) and influenza viruses and (2) an Addavax only control. All mice, regardless of vaccination strategy (see Fig 1), were serially phlebotomized on days 0, 21, 42 and 63, and sera tested individually by mPlex-Flu analysis to assess cross-reactive antibodies.

**Fig 5:**
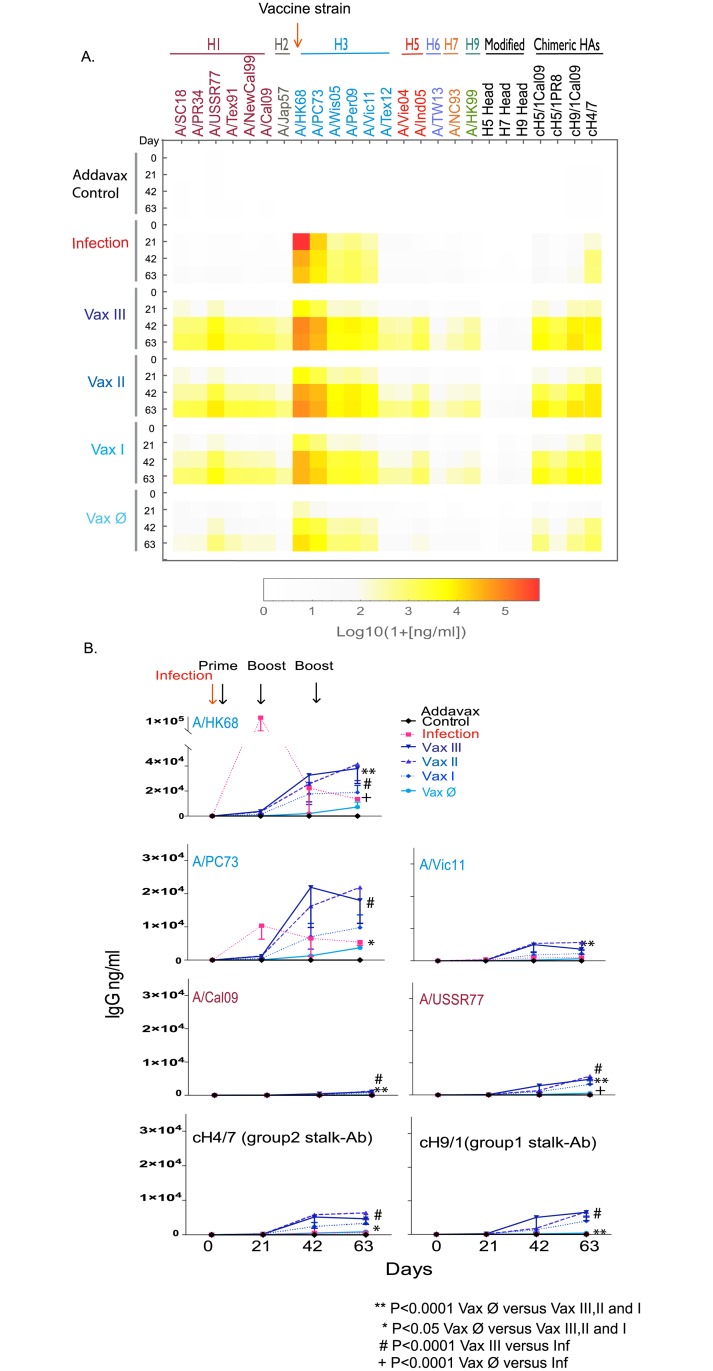
Broader cross-reactive antibody responses are induced by prime-boost-boost vaccination with Addavax adjuvanted rHA as compared to infection with A/Hong Kong/1/1968 (A/HK68 H3N2) influenza virus. (**A**). The rHA specific IgG concentrations of heterosubtypic and stalk-specific antibodies reaction induced by vaccination with adjuvant alone control, rHA combined with Addavax adjuvant (Vax I, II and III), rHA without adjuvant (Vax Ø), or infection (Inf) with A/HongKong/1/68 (H3N2) virus, were determined using a 29-panel mPlex-Flu assay, and represented as a heatmap. (**B**). The selected antibody responses against influenza virus HA of A/HK68 were plotted over time. The antibody concentrations (ng/mL) of individual HA were calculated from the standard curves generated in same assay. Serum of individual mice were harvested before priming, as well as before and post-boost 21 day. The specific antibody concentration of IgG is shown as the mean ± standard deviations (STD) of the mean of individual mice (n = 6). The two-way analysis of variance (ANOVA) statistical analysis was conducted including experimental group and time as factors. ** p<0.0001 Vax Ø comparing to Vax III, II and I group; * p<0.05 Vax Ø comparing to Vax I, II, III group; # p<0.0001 Vax III comparing to Inf group; + p<0.0001 Vax comparing to Inf group.

The results indicate that priming with Addavax adjuvanted rHA of A/HK68 vaccine is the key step for inducing broad cross-reaction (Fig 5 and S6 Fig). We observed statistically significant increases in cross-reactivity against all H3 subtypes we tested in groups primed with adjuvanted rHA of A/HK68 virus (Vax I, II, and III) as compared to the non-adjuvanted rHA group (Vax Ø) and adjuvant alone control (Fig 5) (p<0.0001). This increased cross-reactivity was seen 21 days after the priming vaccination, and continued to increase after each of two subsequent boosting vaccinations, 42 and 63 days after priming (Fig 5A and 5B). There was no significant difference at any time point between any of the groups that received an adjuvanted rHA priming vaccine (Vax I, II, and III), irrespective of the inclusion of Addavax adjuvant in subsequent boosting vaccinations. Similar results were observed in the cohort primed with adjuvanted A/Cal09 rHA (Panels A and B in S6), which had a statistically significant increase (p<0.0001) in broadly cross-reactive antibodies against the H1 subtype HAs, along with weak cross-reactive antibodies to H5 HAs. These data strongly suggest that Addavax adjuvant in primary immunization with rHA proteins is necessary for induction of broad cross-reactive response and, significantly, it may not be necessary to admix rHA with oil-in-water adjuvant in subsequent boosting vaccinations. These results are consistent with the recently published clinical MF59 adjuvanted H7 vaccination study [41].

These experiments also revealed that sequential boosting vaccinations with rHA alone at 21 and 42 days post-priming significantly increased the breadth and magnitude of vaccine strain specific and cross-reactive anti-HA IgG in all four vaccination groups. This was true even in mice vaccinated with rHA alone (Vax Ø), although the anti-HA IgG levels were lower than those produced by adjuvanted vaccinations. Of note, while boosting with rHA protein twice is necessary for induction of both of specific and broadly cross-reactive anti-HA IgG in mice, the addition of adjuvant in the boosting step is not as critical as the presence of adjuvant during priming.

### Stalk-reactive antibodies contribute to cross-reactivity induced by multiple adjuvanted rHA vaccinations in mice

We next asked if cross-reactivities induced by an adjuvanted rHA prime-boost-boost vaccination strategy were due to increased levels of anti-HA stalk reactive IgG antibodies. In order to evaluate the concentration of group 1 and 2 stalk-reactive antibodies, we measured post-vaccination IgG binding to chimeric HAs (cHA) [18, 21, 42]. Compared to homologous viral infection, rHA vaccination with A/HK68 significantly increased the concentration of group 2 stalk-reactive antibodies binding to the cH4(head)/H7(stalk) cHA in all experimental groups (Vax I, II, III and Vax Ø) (Fig 5A and 5B). Similar results were found in the A/Cal09 HA vaccination cohort, where vaccination with rHA induced stronger stalk-reactive IgG against group 1 stalk HA, as measured by serum binding to cH9(head)/H1(stalk, A/Cal09) cHAs (Panels A and B in S6 Fig). We specifically examined reactivity against the cH9/1_Cal09_ to evaluate the group 1 stalk-reactive antibodies given that vaccination against A/Cal09 rHA also induced cross-reactive antibodies against H5 rHAs. These increased stalk-reactive IgG antibody levels correlated well with the overall broad cross response induced by rHA vaccination (i.e. measurement of post-vaccination serum IgG binding to non-chimeric rHA), as described above. This finding suggests that adjuvanted rHA vaccine-induced stalk-reactive antibodies most likely contribute to the IgG cross-binding to different influenza strains, subtypes, and phylogenetic groups. These findings are consistent with other studies showing the contribution of stalk-reactive antibodies to cross-reactivity in humans and mice (reviewed in [14]).

### Priming and boosting with adjuvanted rHA protein increased bone marrow resident antibody secreting cells (ASCs) producing broadly cross-reactive anti-HA IgG

**Fig 6:**
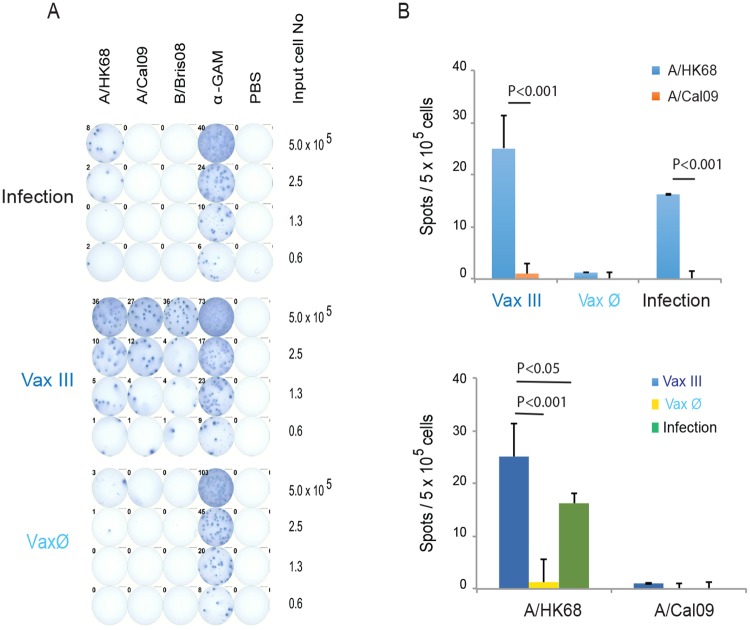
The cross-reactive anti-HA IgG secreting plasma cells (ASCs) in murine bone marrow after infection or vaccination with A/HongKong/1/1968 (A/HK68 H3N2) influenza virus. **(A)** Representative plates of ELISpot assays as described in the methods, which evaluated antibody secreting plasma cells in murine bone marrow specific against rHA of A/California/07/2009 (A/Cal09, H1N1), A/HongKong/1/1968 (A/HK68, H3N2) and B/Bris08 (B strain) influenza viruses induced by vaccination with Addavax adjuvanted rHA of A/HK68 (VaxIII), without adjuvant (Vax Ø) and by A/HK68 virus infection group. **(B)** Representative numbers of IgG ASCs specific for rHA of A/HK68 and A/Cal09 starting with 500,000 murine bone marrow cells, after subtracting the numbers of ASC spots specific for rHA of B/Bris08 (background control), induced after vaccination (VaxIII, Vax Ø groups) or infection. The values and error bars shown are the mean and the standard deviation (STD) of n = 4–5 mice/time point. Grouped multiple t-tests were used to determine statistically significant differences.

The presence of CD138^+^, bone marrow resident plasma cells post-vaccination is critical for long-lived IgG mediated immunity [30]. Murine CD138^+^ plasma cells (Ab secreting cells, ASCs) home to and engraft within the bone marrow approximately 45 days after influenza vaccination or infection [30]. In order to assess the post-vaccination presence of influenza virus HA specific ASCs in bone marrow that produce cross-reactive antibodies, we harvested bone marrow mononuclear cells from mice after A/HK68 viral infection or rHA vaccination with (Vax III) and without Addavax adjuvant (Vax Ø), 21 days after second boost (63 days after priming). We then measured cross-strain IgG reactivity to rHA of A/Cal09, A/HK68 and influenza B virus HA by ELISpot, as previously described [43]. The anti-IgG capture antibody coated wells showed all ASCs as a positive control; wells coated with rHA of influenza B/Bris08 were used as an additional control for any potential background arising from antibodies to the TTH6 thrombin cleavage site, trimerization domain or hexahistidine tag regions. Compared to infection and non-adjuvanted vaccine groups (Vax Ø), the adjuvanted rHA vaccine mice (Vax III) had significantly more broadly cross-reactive, bone marrow resident, ASCs (Fig 6, p<0.001). These data support our serological findings, and suggest that the cross-reactive antibody response detected by the mPlex-Flu assay correlates with the presence of bone marrow resident ASCs producing homogenously or heterogeneously reactive antibodies.

### Priming and boosting with Addavax adjuvanted rHA elicited splenic memory B cells (MBCs)

Long-lived memory B cells (MBCs) are generated concurrently with vaccine-specific ASCs during B cell vaccine responses. Upon reactivation, these MBCs will divide and differentiate into ASCs to mediate a rapid and vigorous secondary antibody response [22]. LPS has been shown to non-specifically stimulate MBCs [30], while BPL-inactivated influenza viruses stimulate virus-specific MBCs in mice [31].

**Fig 7:**
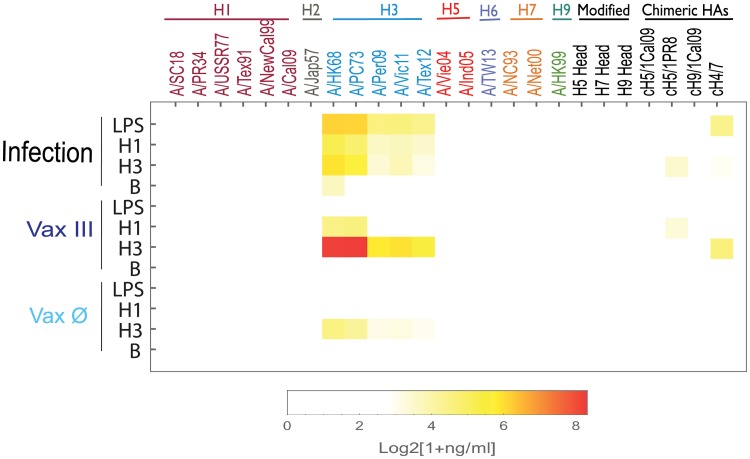
Comparison of anti-HA IgG from mouse spleen memory B cells (MBC) stimulated by infection with A/HongKong/1/1968 (A/HK68 H3N2) virus or vaccinated with adjuvanted rHA. The anti-HA IgG levels in supernatants of stimulated memory B cells (MBC) cultures were determined by 29-plex panel mPlex-Flu assay. Briefly, mouse spleen MBCs were cultured for 6 days with 0.4 μg LPS (LPS), 10 μg BPL-inactivated A/Cal09 virus (H1) and B/Brisbane/60/2008 virus (B), 2009–2013 H3N2 viral antigen (H3), and medium control (Con), respectively. Influenza virus specific antibody concentrations present in the cell culture supernatants are shown as the mean of IgG concentrations after subtracting the control baseline antibodies in control unstimulated cell cultures (n = 3–4) and represented as a heatmap. The values of IgG concentrations without subtracting the control baseline antibodies in unstimulated cultures (Con), shown in Supplementary material (S7 Fig), demonstrate that only a low level of IgG antibodies produced by murine splenic B cells, after infection and vaccination, without stimulation.

In order to evaluate the effects of adjuvant on the MBC responses against cross-strain HA, we collected spleen mononuclear cells (an abundant source of MBCs[30]) of mice after infection with A/HK68, as well as after vaccination against A/HK68 rHA with (Vax III) and without (Vax Ø) Addavax adjuvant at 21 days after the last boost (63 days after primary influenza virus infection or primary rHA vaccination). Those splenic cells were cultured and left unstimulated control (Con) or were stimulated *in vitro* with LPS or BPL-inactivated H3N2 viruses and supernatants analyzed after 6 days for IgG against a panel of 31 rHAs. The results (S7 Fig) revealed low levels of influenza antibodies produced by ASCs in culture supernatants of unstimulated splenic cells in both infection and vaccination groups. Subtracting the baseline control after stimulation (Fig 7), the anti-HA IgG produced by MBCs in the adjuvanted vaccination group (Vax III) showed broader cross-strain HA binding activity compared to stimulated MBC from the infection alone group (p <0.01) which only showed cross-reactivity within the H3 subtype. Overall, *in vitro* stimulated MBCs from mice that received the adjuvanted vaccine produced significantly higher levels of IgG against homologous and heterologous HAs of H3 viruses when compared to MBCs from the viral infection group (Fig 7). We also observed cH4/7 reactive IgG antibodies after stimulation with H3, suggesting that IgG cross reactivity was generated against the H7 stalk. Of note, influenza specific MBCs from Addavax adjuvanted H3 rHA vaccinated mice stimulated with H1 virus also produced cross-reactive IgG antibodies against historical H3 strains A/HK68 and A/PC73. These data suggest that the strategy of adjuvanted prime-boost-boost vaccination with rHA of A/HK68 increases H3 specific MBCs, and their ability to produce cross-reactive and stalk-reactive IgG antibodies in a subsequent recall response in mice.

### Sequential vaccination with Addavax adjuvanted rHA does elicit broader cross-neutralization against heterovariant influenza virus infection from the same phylogenic group

We next assessed if the broadly reactive anti-rHA IgG induced by Addavax rHA vaccination correlated with higher levels of neutralizing antibodies capable of blocking viral infection. We measured the viral neutralization activity of mouse antisera generated from Addavax adjuvanted rHA vaccination against both vaccine specific and heterologous influenza viruses *in vitro* using a plague formation based microneutralization assay[33].

We found that the mouse sera generated by multiple Addavax adjuvanted A/HK68 rHA protein vaccinations effectively neutralized homologous A/HK68 virus, and also cross-neutralized the A/Per09 virus, preventing *in vitro* infection (Table 3). Although A/Per09 is an H3 influenza virus within the same subtype as A/HK68, A/Per09 exhibits significant antigenic drift as compared with A/HK68 (S3 Fig). These results correlate with our above observation that an adjuvanted rHA multiple vaccination strategy induces broader anti-HA IgG cross-reactivity than infection. This finding is also consistent with clinical studies showing that squalene-based-oil-in-water adjuvanted influenza vaccination induced broadly reactive neutralizing antibodies in human subjects [25, 33]. Interestingly, we observed that anti-HA antibodies in sera generated with adjuvanted A/Cal09 rHA vaccination reproducibly showed weak cross-reactivity to the heterologous A/Per09 virus (H3) compared to that induced by infection, despite the fact that the homologous (anti-H1) serum neutralization titers were similar. In addition, we used an FFU-based assay to evaluate antisera of infection and vaccination, which independently confirmed these results (S1 Table). A sequence dissimilarity dendrogram analysis demonstrates that HA of A/Cal09 virus is more similar to H3 influenza viruses than other H1 viruses. Also, this finding is consistent with clinical studies showing that MF59 adjuvanted influenza vaccination induces broadly reactive neutralizing antibodies in human subjects [25, 33].

**Table 3:** The neutralization titers (GMT) of antisera generated by viral infection or Addavax adjuvanted rHA vaccination of A/Cal09(H1) or A/HK68 (H3) influenza.

Groups	Tested influenza viruses				
	A/Cal09 (H1)	A/PR8 (H1)	A/HK68 (H3)	A/Per09 (H3)	B/Bris08 (B)
Addavax Control	≤ 50	≤ 50	≤ 50	≤ 50	≤ 50
A/Cal09 Infection	4,031	≤ 50	≤ 50	≤ 50	≤ 50
Addavax + rHA A/Cal09	6,063	≤ 50	≤ 50	283*	≤ 50
A/HK68 Infection	≤ 50	≤ 50	5,080	63	≤ 50
Addavax + rHA A/HK68	≤ 50	≤ 50	6,400	503*	≤ 50

* P< 0.001 compared to infection group in same cohort.

## Discussion

A major recent objective of influenza vaccine development is to develop clinical strategies that induce broadly cross-reactive and protective anti-influenza antibodies. Increasing data suggest that IgG antibodies targeting the conserved influenza HA stalk protein region display broader cross-reactivity and higher neutralizing potential across several influenza strain subtypes, including between group 1 and group 2 influenza viruses [18, 21, 42]. Currently, the specific mechanisms responsible for the induction of protective cross-reactive and stalk-reactive IgG antibodies are not well characterized. Two empirically successful strategies have been identified: sequential immunization with either (1) stabilized stalk domain [9, 10] or (2) cHA proteins or virus containing the identical stalk domains but different head regions [19, 21, 44]. The current working hypotheses are that boosting twice after priming may increase the production of stalk-reactive antibodies, and that B cell exposure to the same stalk domain with different head domains using cHAs breaks the immunodominant recognition of the influenza head region in the germinal center reaction, increasing production of stalk-reactive IgG antibodies (reviewed in [14]).

Our primary finding is that a multiple vaccination strategy with squalene-in-oil adjuvanted rHA induces broad IgG mediated cross-reactivity to heterologous influenza virus HA(Figs 2 and 3). Significantly, the addition of the MF59-like adjuvant Addavax in the priming stage of a triple vaccination schedule (prime-boost-boost) was necessary and sufficient for eliciting broadly cross-reactive anti-influenza HA responses for inducing both IgG mediated cross-reactivity, as well as expanding broadly cross-reactive memory B cells that can mount a vigorous recall response when exposed to heterologous influenza virus HA (Fig 5).

Our work demonstrates that a prime-boost-boost vaccination strategy with adjuvanted rHA protein elicits cross-reactive IgG antibodies against both influenza virus HA homologous to the vaccine strain, as well as other heterologous and heterosubtypic HAs in ferrets (Freunds adjuvant) and mice (MF59-like squalene adjuvant). Our results also demonstrate that stalk-reactive antibodies contribute significantly to the observed cross-reactivities, as detected by binding of post-vaccination serum IgG to cHAs (cH9/1 and cH4/7). Importantly, we found that adjuvanted vaccination in mice robustly enhances the numbers of bone marrow resident plasma cells (Fig 6) and class-class switched splenic memory B cells capable of producing broadly cross-reactive anti-HA IgG with increased cross-strain HA binding (Fig 7). Compared to intramuscular vaccination with inactivated influenza virus, non-adjuvanted vaccination with rHA protein generally produces weaker anti-influenza vaccine-specific IgG responses, even with a prime-boost-boost strategy [21]. To our knowledge, this is the first report showing that a prime-boost-boost vaccination strategy with soluble recombinant HA protein and a squalene-based-oil-in-water adjuvant can efficiently induce broadly cross-reactive stalk-reactive IgG antibodies as compared to a single influenza viral infection.

Our work may also be relevant to the current debate regarding the role of “original antigenic sin” or “imprinting” in influenza virus vaccine responses [45–48]. This creates an immune bias towards responses against strains that an individual has been previously exposed to, and may blunt B cell responses against antigenically dissimilar HAs. The mechanisms for this are currently not well characterized, but one hypothesis is that the strong response of memory B cells, augmented by T follicular helper cells (TfH), may out-compete responses to new influenza virus strain antigens [49]. The adjuvant MF59 has been reported to induce strong TfH responses to influenza vaccination, activate a large pool of distantly primed memory B cells[50], and appears to result in long-lived, protective cross-reactive antibodies [24, 25, 40, 51, 52]. As such, recent work has shown that influenza virus vaccine responses may depend critically on existing anti-HA immunity from prior influenza infection and/or vaccination, which is very hard to assess using any of the traditional single dimensional assays, such as HAI, MN and ELISA. However, our mPlex-Flu assay allows for the efficient assessment of antibody responses covering HAs of all previous and current circulating and vaccine strains. Such a method provides a high-throughput and quantitative estimate of the imprinting pattern for each subject pre- and post-vaccination. Our results provide additional and detailed support that a primary adjuvanted influenza rHA vaccination can induce broadly cross-reactive, IgG class switched B cells, which are persistent and respond to further boosting from either adjuvanted or non-adjuvanted vaccinations. One important caveat is that our experiments were performed in HA-naïve mice. This condition is generally not found in human subjects, where prior multiple vaccinations and infections have occurred. Thus, further validation of these findings in multiply vaccinated or exposed mice, or in human population level studies, should be performed.

Another potential caveat to this work relates to the mouse vaccine experiments, where we used a hybrid rHA-TT6H protein, containing C-terminal trimerization domain and His-tag, for vaccination. This approach, however, allowed us to use mouse adapted A/Cal/07/09 influenza strains in further microneutralization experiments, comparing homologous and cross-strain neutralization after infection versus vaccination. Importantly, despite the potential for detecting “cross-reactive” antibodies in the mPlex-flu assay related to anti-TTH6 IgG, extensive control experiments demonstrated that such binding is significantly “silenced” after the rHAs are coupled to Luminex beads via C-terminal specific binding. This is most likely caused by steric blocking of the trimerization domain and His-tag by the large HA head and/or stalk domains, similarly to what happens to the membrane-proximal sequences of the HA stalk domain on the influenza virus surface [4]. Despite observing decreased binding of trimerization domain and His-tag antibodies in our mPlex-Flu assay, however, we did not observe a significant decrease in the detection of stalk-reactive antibodies, as verified by binding of both the monoclonal stalk-reactive antibodies, 1B11 and KB2 [53] (S2 Fig). Perhaps most importantly, results from the murine vaccine experiments were highly consistent with our findings in the ferret vaccination sera generated with rHA that did not contain TTH6 sequences.

The *in vivo* murine challenge model is commonly used for estimating protective antibody levels against particular influenza virus strains. However, there are a very limited number of mouse-adapted influenza virus strains available for testing broad cross-protection in mice. In addition, some studies showing enhancement of protection of anti-stalk antibody against influenza viruses using *in vivo* challenging assays in mouse models, do not always result in increased neutralizing activity against the same viral strains *in vitro* microneutralization assays[12, 13]. These results are most likely due to *in vivo* Fc-mediated antibody dependent cellular cytotoxicity (ADCC) and complement dependent lysis (CDL), which are not measured by *in vitro* neutralization assays [12, 13]. Despite these model and assay limitations, we attempted to address whether the cross-reactive antibodies detected by our mPlex-Flu assay have functional relevance, we performed a fluorescence-forming units (FFU) quantified multiple life-cycle microneutralization assay. This assay has the benefit of directly measuring the number of virus-infected cells to normalize the viral input of different influenza viruses, which is particularly useful for viruses that induce different cytopathic effects and for viruses such as influenza whose plaques do not stick to the agar well enough to be accurately quantified by a plaque assay. Using this assay, we were able to detect weakly cross-protective antibody activity against H3 influenza virus (A/ Per09) in the serum induced by both of adjuvanted A/HK68 and A/Cal09 vaccination with rHA and infection of virus (Table 3).

Taken together, our findings suggest that the use of squalene-based oil-in-water adjuvanted rHA influenza vaccines may be highly desirable. Such a strategy may induce population level priming, providing some protection against emerging influenza viruses that have undergone antigenic shift. Our findings complement recent work showing that Addavax, an MF59-like adjuvant, significantly enhances IgG mediated reactivity against heterologous strains of influenza in humans, and elicits a memory B cells (MBCs) pool that is capable of recall responses producing strong cross-reactive protection against H5 influenza viruses after booster vaccination [24, 54, 55]. While the mechanisms for such enhancement remain to be characterized, other evidence suggests that adjuvanted influenza virus vaccine promotes affinity maturation of antibodies in a prime-boost-boost strategy [56]. The data from our current study supports the hypothesis that use of a squalene-based oil-in-water adjuvant during the primary vaccination is critical for inducing cross-strain anti-HA IgG responses, but does not significantly increase cross-reactivity in subsequent boosting vaccination with homologous strains. Current MF59 adjuvanted influenza vaccine formulations are only approved in the United States for individuals older than 65 years. Our findings may be helpful for future adjuvanted vaccination strategy design.

## Supporting information

S1 File Single circle fluorescent forming units (FFU) based neutralization assay. Methods and results of single circle fluorescent forming unit based microneutralization assays. (S1 Table).
(DOCX)

S1 Table Serum neutralization titers induced after viral infection or Addavax Adjuvanted rHA vaccination with A/Cal09(H1) or A/HK68 (H3) influenza virus strains. Neutrilzation assays were performed against against A/HK68, A/Cal09, A/Puerto Rico/8/1934 (A/PR8) and B/Bris08 influenza strains. Sera from the following groups were compared: Addavax Control, A/Cal09 Infection, rHA A/Cal09 + Addavax, A/HK68 Infection, and rHA A/HK68 + Addavax. Results demonstrated significantly higher titers that neutralized homologous and cross-strain influenza virus.
(DOCX)

S1 Fig Purified recombinant hemagglutinin (rHA) proteins of influenza viruses. The ectodomain of HA0 genes of influenza viruses were expressed in the baculovirus system using a modified pFastBac vector (Invitrogen) that harbors a hexahistidine tag at the C-terminal. The rHAs were purified with Ni-NTA resin (Qiagen) and concentrated with Amicon Ultra 30K filters (Millipore). **A**. The SDS-PAGE gel of rHAs preparations. The purified rHAs were separated and analyzed by 4–12% Bis-Tris NuPage gels (Invitrogen). **B**. The antigenic characteristics of rHAs of influenza virus were verified by the e Western-blot analysis of rHAs preps. Purified recombinant HAs were separated on SDS-PAGE and transferred to nitrocellulose membrane (0.45μm, Bio-Rad). After the membrane was blocked with 5% milk in TBST buffer (500 mM NaCl, 20 mM pH 7.5 Tris–HCl, 0.05% Tween-20)) for 30min at room temperature, it was detected with ferret polyclonal antisera of influenza A subtype viruses provided by Influenza Reagent Resource (IRR) **1)** antiserum to A/California/04/2009(H1N1) (FR-359); **2)** antiserum to A/Perth/16/2009 (H3N2) (FR-446); **3)** antiserum to A/Japan/305/1957 (H2N2) (FR-891), A/Vietnam/1203/2004 (H5N1) (FR-708), A/Netherlands/219/2003 (H7N7) (FR-890) and A/HongKong/1073/1999 (H9N2) (FR-889).
(PDF)

S2 Fig Verification of mPlex-Flu assay with monoclonal antibodies against type A influenza viruses and stalk-domain of HA of group 1 type A influenza viruses. The mPlex-Flu assay was tested using mouse monoclonal antibodies to HA of H1, H3 and flu B influenza virus from Influenza Reagent Resource (IRR) and anti-stalk monoclonal antibodies 1B11 and KB2 from Dr. Krammer. All antibodies were diluted to 2.5 μg/mL final concentration, and PE conjugated anti-human IgG (γ chain specific) secondary antibodies was 1:400 diluted. The results are the mean median fluorescence intensity (MFI) subtracting the blank of each influenza virus strain from three replicate wells.
(EPS)

S3 Fig The protein sequence distance calculation. Sequence dissimilarity of HA molecular based on the protein sequences of rHAs using Euclidean distance measurement and protein feature vector method were performed to estimate the distance between the actual and theoretical sequence based on the binomial and uniform distributions [34, 35], and metric multidimensional scaling was performed using custom Mathematica code.
(PDF)

S4 Fig The ectodomain of recombinant HA0 could not be stabilized after cleavage of the trimerization domain from the fusion A/California/07/2009 rHA protein preparations. Recombinant ectodomain HA0 of A/California/07/2009 was cleaved off from the His-tagged fusion protein by thrombin treatment at room temperature for 4 hours at room temperature. Then the ectodomain rHA protein preps were sampled after 4, 18 and 30 hours at 4 degrees, and they were analyzed by SDS-PAGE gel.
(EPS)

S5 Fig Coupling rHA of influenza viruses “silence” the antigenicity of C-terminal His-tag and trimerization domain. A 46aa peptide (TT6H) consisting of a thrombin cleavage site, a trimerization domain from bacteriophage T4 fibritin protein (Fd) and hexahistidine tag, sharing the same protein sequence with the C-terminus of expressed and purified rHAs was synthesized. Then TT6H and rHAs of influenza viruses were coupled onto Luminex beads in the same molar concentration and conditions. The hexahistine tagged specific antibodies and Fd specific antibodies were tested by using TT6H coupled beads mixed with other rHAs coupled beads reaction with mouse monoclonal anti-C terminal hexahistidine tag antibody, a rabbit polyclonal anti-trimerization Fb domain antisera, and sera from ferrets infected or immunized with A/Cal09 (H1N1) influenza virus or A/Cal09 rHA, respectively, in working dilutions. Then subtracted the MFI with data from naïve animal sera in same dilutions to normalize data. The heat map of mean MFI was generated from two duplicates and two independent experiments.
(EPS)

S6 Fig Broader cross-reactive antibody response was induced by prime-boost-boost vaccination with adjuvanted rHA versus infection with A/Cal09 (H1N1) Influenza virus. (**A**). The heatmap of IgG concentrations of cross-reactive antibodies and stalk-specific serum antibodies induced by vaccination with rHA with or without Addavax (MF59-like) adjuvant or infection with A/Cal09 (H1N1) were determined using a 29-panel mPlex-Flu assay. The antibody binding reactions (MFI units) were converted into the concentration of antibodies (ng/mL) using the standards curves generated in same assay. The tested sera were harvested before priming, and before and after every boost. The specific antibody concentration of IgG shows the means of six mice in the same group. (**B**). The selected individual homologous and heterologous antibody responses were plotted over time from the above data. The values and error bars shown are means and standard deviations (STD) of the mean of six mice. The two-way analysis of variance (ANOVA) statistical analyses was conducted including experimental group and time as factors. ** P<0.0001 Vax comparing to Vax III, II and I; * P<0.05 Vax comparing to Vax III, II and I; # P<0.0001 Vax III comparing to Infection; + P<0.0001 Vax comparing to Infection.
(EPS)

S7 Fig Influenza antibodies produced by spleen memory B cells (MBC) stimulated by influenza viruses in the mice infected by A/Hong Kong/1/1968 (A/HK68 H3N2) virus or vaccinated with adjuvanted rHA. The antibody levels in supernatants from memory B cells (MBC) cultures were determined by 29-plex panel mPlex-Flu assay as described in the experiment methods. The heatmap of antibody concentrations shown are the mean of IgG concentrations (n = 3–4).
(EPS)

S8 Fig The influenza antibodies produced by spleen memory B cells (MBC) stimulated with influenza viruses in the mice infected with A/California/07/2009 (H1N1) virus or vaccinated with adjuvanted rHA. The antibody levels in supernatants from memory B cells (MBC) cultures were determined by 29-plex panel mPlex-Flu assay as described in the experiment methods. The heatmap of antibody concentrations of influenza virus in the supernatants are shown as the mean of IgG concentration subtracting the baseline antibodies in unstimulated cell cultures (n = 3–4).
(EPS)

## References

[1] Jayasundara K, Soobiah C, Thommes E, Tricco AC, Chit A. Natural attack rate of influenza in unvaccinated children and adults: a meta-regression analysis. BMC Infect Dis. 2014;14:670 doi: 10.1186/s12879-014-0670-5 . 25495228 10.1186/s12879-014-0670-5 PMC4272519

[2] Guo H, Baker SF, Martinez-Sobrido L, Topham DJ. Induction of CD8 T cell heterologous protection by a single dose of single-cycle infectious influenza virus. Journal of virology. 2014;88(20):12006–16. doi: 10.1128/JVI.01847-14 . 25100831 10.1128/JVI.01847-14 PMC4178714

[3] Fiore AE, Uyeki TM, Broder K, Finelli L, Euler GL, Singleton JA, et al Prevention and control of influenza with vaccines: recommendations of the Advisory Committee on Immunization Practices (ACIP), 2010. MMWR Recomm Rep. 2010;59(RR-8):1–62. . 20689501

[4] Sui J, Hwang WC, Perez S, Wei G, Aird D, Chen LM, et al Structural and functional bases for broad-spectrum neutralization of avian and human influenza A viruses. Nat Struct Mol Biol. 2009;16(3):265–73. doi: 10.1038/nsmb.1566 . 19234466 10.1038/nsmb.1566 PMC2692245

[5] Heaton NS, Sachs D, Chen CJ, Hai R, Palese P. Genome-wide mutagenesis of influenza virus reveals unique plasticity of the hemagglutinin and NS1 proteins. Proceedings of the National Academy of Sciences of the United States of America. 2013;110(50):20248–53. doi: 10.1073/pnas.1320524110 . 24277853 10.1073/pnas.1320524110 PMC3864309

[6] Gerdil C. The annual production cycle for influenza vaccine. Vaccine. 2003;21(16):1776–9. Epub 2003/04/11. . 12686093 10.1016/s0264-410x(03)00071-9

[7] DeDiego ML, Anderson CS, Yang H, Holden-Wiltse J, Fitzgerald T, Treanor JJ, et al Directed selection of influenza virus produces antigenic variants that match circulating human virus isolates and escape from vaccine-mediated immune protection. Immunology. 2016;148(2):160–73. doi: 10.1111/imm.12594 . 26854888 10.1111/imm.12594 PMC4863573

[8] Laurie KL, Engelhardt OG, Wood J, Heath A, Katz JM, Peiris M, et al International Laboratory Comparison of Influenza Microneutralization Assays for A(H1N1)pdm09, A(H3N2), and A(H5N1) Influenza Viruses by CONSISE. Clinical and vaccine immunology: CVI. 2015;22(8):957–64. doi: 10.1128/CVI.00278-15 . 26108286 10.1128/CVI.00278-15 PMC4519725

[9] Impagliazzo A, Milder F, Kuipers H, Wagner MV, Zhu X, Hoffman RM, et al A stable trimeric influenza hemagglutinin stem as a broadly protective immunogen. Science. 2015;349(6254):1301–6. doi: 10.1126/science.aac7263 . 26303961 10.1126/science.aac7263

[10] Yassine HM, Boyington JC, McTamney PM, Wei CJ, Kanekiyo M, Kong WP, et al Hemagglutinin-stem nanoparticles generate heterosubtypic influenza protection. Nat Med. 2015;21(9):1065–70. doi: 10.1038/nm.3927 . 26301691 10.1038/nm.3927

[11] Ekiert DC, Kashyap AK, Steel J, Rubrum A, Bhabha G, Khayat R, et al Cross-neutralization of influenza A viruses mediated by a single antibody loop. Nature. 2012;489(7417):526–32. doi: 10.1038/nature11414 . 22982990 10.1038/nature11414 PMC3538848

[12] Dreyfus C, Laursen NS, Kwaks T, Zuijdgeest D, Khayat R, Ekiert DC, et al Highly conserved protective epitopes on influenza B viruses. Science. 2012;337(6100):1343–8. doi: 10.1126/science.1222908 . 22878502 10.1126/science.1222908 PMC3538841

[13] DiLillo DJ, Tan GS, Palese P, Ravetch JV. Broadly neutralizing hemagglutinin stalk-specific antibodies require FcgammaR interactions for protection against influenza virus in vivo. Nat Med. 2014;20(2):143–51. doi: 10.1038/nm.3443 . 24412922 10.1038/nm.3443 PMC3966466

[14] Krammer F. Novel universal influenza virus vaccine approaches. Current opinion in virology. 2016;17:95–103. doi: 10.1016/j.coviro.2016.02.002 . 26927813 10.1016/j.coviro.2016.02.002 PMC4902764

[15] Nguyen HH, van Ginkel FW, Vu HL, McGhee JR, Mestecky J. Heterosubtypic immunity to influenza A virus infection requires B cells but not CD8+ cytotoxic T lymphocytes. J Infect Dis. 2001;183(3):368–76. doi: 10.1086/318084 . 11133367 10.1086/318084

[16] Tumpey TM, Renshaw M, Clements JD, Katz JM. Mucosal delivery of inactivated influenza vaccine induces B-cell-dependent heterosubtypic cross-protection against lethal influenza A H5N1 virus infection. Journal of virology. 2001;75(11):5141–50. doi: 10.1128/JVI.75.11.5141-5150.2001 . 11333895 10.1128/JVI.75.11.5141-5150.2001 PMC114919

[17] Epstein SL, Lo CY, Misplon JA, Lawson CM, Hendrickson BA, Max EE, et al Mechanisms of heterosubtypic immunity to lethal influenza A virus infection in fully immunocompetent, T cell-depleted, beta2-microglobulin-deficient, and J chain-deficient mice. J Immunol. 1997;158(3):1222–30. . 9013963

[18] Hai R, Krammer F, Tan GS, Pica N, Eggink D, Maamary J, et al Influenza viruses expressing chimeric hemagglutinins: globular head and stalk domains derived from different subtypes. J Virol. 2012;86(10):5774–81. doi: 10.1128/JVI.00137-12 . 22398287 10.1128/JVI.00137-12 PMC3347257

[19] Krammer F, Pica N, Hai R, Margine I, Palese P. Chimeric hemagglutinin influenza virus vaccine constructs elicit broadly protective stalk-specific antibodies. J Virol. 2013;87(12):6542–50. doi: 10.1128/JVI.00641-13 . 23576508 10.1128/JVI.00641-13 PMC3676110

[20] Margine I, Krammer F, Hai R, Heaton NS, Tan GS, Andrews SA, et al Hemagglutinin stalk-based universal vaccine constructs protect against group 2 influenza A viruses. J Virol. 2013;87(19):10435–46. doi: 10.1128/JVI.01715-13 . 23903831 10.1128/JVI.01715-13 PMC3807421

[21] Goff PH, Eggink D, Seibert CW, Hai R, Martinez-Gil L, Krammer F, et al Adjuvants and immunization strategies to induce influenza virus hemagglutinin stalk antibodies. PloS one. 2013;8(11):e79194 doi: 10.1371/journal.pone.0079194 . 24223176 10.1371/journal.pone.0079194 PMC3819267

[22] Ahmed R, Gray D. Immunological memory and protective immunity: understanding their relation. Science. 1996;272(5258):54–60. . 8600537 10.1126/science.272.5258.54

[23] Wack A, Baudner BC, Hilbert AK, Manini I, Nuti S, Tavarini S, et al Combination adjuvants for the induction of potent, long-lasting antibody and T-cell responses to influenza vaccine in mice. Vaccine. 2008;26(4):552–61. doi: 10.1016/j.vaccine.2007.11.054 . 18162266 10.1016/j.vaccine.2007.11.054

[24] Galli G, Hancock K, Hoschler K, DeVos J, Praus M, Bardelli M, et al Fast rise of broadly cross-reactive antibodies after boosting long-lived human memory B cells primed by an MF59 adjuvanted prepandemic vaccine. Proceedings of the National Academy of Sciences of the United States of America. 2009;106(19):7962–7. Epub 2009/05/07. doi: 10.1073/pnas.0903181106 . 19416838 10.1073/pnas.0903181106 PMC2674105

[25] Fragapane E, Gasparini R, Schioppa F, Laghi-Pasini F, Montomoli E, Banzhoff A. A heterologous MF59-adjuvanted H5N1 prepandemic influenza booster vaccine induces a robust, cross-reactive immune response in adults and the elderly. Clin Vaccine Immunol. 2010;17(11):1817–9. doi: 10.1128/CVI.00461-09 . 20810680 10.1128/CVI.00461-09 PMC2976095

[26] Wang J, Hilchey SP, Hyrien O, Huertas N, Perry S, Ramanunninair M, et al Multi-Dimensional Measurement of Antibody-Mediated Heterosubtypic Immunity to Influenza. PloS one. 2015;10(6):e0129858 doi: 10.1371/journal.pone.0129858 . 26103163 10.1371/journal.pone.0129858 PMC4478018

[27] Krammer F, Margine I, Tan GS, Pica N, Krause JC, Palese P. A carboxy-terminal trimerization domain stabilizes conformational epitopes on the stalk domain of soluble recombinant hemagglutinin substrates. PloS one. 2012;7(8):e43603 doi: 10.1371/journal.pone.0043603 . 22928001 10.1371/journal.pone.0043603 PMC3426533

[28] Sanders RW, van Gils MJ, Derking R, Sok D, Ketas TJ, Burger JA, et al HIV-1 VACCINES. HIV-1 neutralizing antibodies induced by native-like envelope trimers. Science. 2015;349(6244):aac4223 doi: 10.1126/science.aac4223 . 26089353 10.1126/science.aac4223 PMC4498988

[29] He XS, Sasaki S, Narvaez CF, Zhang C, Liu H, Woo JC, et al Plasmablast-derived polyclonal antibody response after influenza vaccination. J Immunol Methods. 2011;365(1–2):67–75. doi: 10.1016/j.jim.2010.12.008 . 21182843 10.1016/j.jim.2010.12.008 PMC3039424

[30] Nduati EW, Ng DH, Ndungu FM, Gardner P, Urban BC, Langhorne J. Distinct kinetics of memory B-cell and plasma-cell responses in peripheral blood following a blood-stage Plasmodium chabaudi infection in mice. PloS one. 2010;5(11):e15007 doi: 10.1371/journal.pone.0015007 . 21124900 10.1371/journal.pone.0015007 PMC2990717

[31] Joo HM, He Y, Sundararajan A, Huan L, Sangster MY. Quantitative analysis of influenza virus-specific B cell memory generated by different routes of inactivated virus vaccination. Vaccine. 2010;28(10):2186–94. doi: 10.1016/j.vaccine.2009.12.058 . 20056191 10.1016/j.vaccine.2009.12.058

[32] Clark AM, Nogales A, Martinez-Sobrido L, Topham DJ, DeDiego ML. Functional Evolution of Influenza Virus NS1 Protein in Currently Circulating Human 2009 Pandemic H1N1 Viruses. Journal of virology. 2017;91(17). doi: 10.1128/JVI.00721-17 . 28637754 10.1128/JVI.00721-17 PMC5553169

[33] Corti D, Suguitan AL Jr., Pinna D, Silacci C, Fernandez-Rodriguez BM, Vanzetta F, et al Heterosubtypic neutralizing antibodies are produced by individuals immunized with a seasonal influenza vaccine. J Clin Invest. 2010;120(5):1663–73. doi: 10.1172/JCI41902 . 20389023 10.1172/JCI41902 PMC2860935

[34] Zand MS, Wang J, Hilchey S. Graphical Representation of Proximity Measures for Multidimensional Data: Classical and Metric Multidimensional Scaling. Math J. 2015;17 doi: 10.3888/tmj.17-7 . 26692757 10.3888/tmj.17-7 PMC4675631

[35] Carr K, Murray E, Armah E, He RL, Yau SS. A rapid method for characterization of protein relatedness using feature vectors. PloS one. 2010;5(3):e9550 doi: 10.1371/journal.pone.0009550 . 20221427 10.1371/journal.pone.0009550 PMC2832692

[36] Quataert SA, Rittenhouse-Olson K, Kirch CS, Hu B, Secor S, Strong N, et al Assignment of weight-based antibody units for 13 serotypes to a human antipneumococcal standard reference serum, lot 89-S(f). Clin Diagn Lab Immunol. 2004;11(6):1064–9. doi: 10.1128/CDLI.11.6.1064-1069.2004 . 15539507 10.1128/CDLI.11.6.1064-1069.2004 PMC524753

[37] Molenberghs G, Verbeke G. Linear Mixed Models for Longitudinal Data. New York: Springer; 2000 334 p.

[38] Krammer F, Pica N, Hai R, Tan GS, Palese P. Hemagglutinin Stalk-Reactive Antibodies Are Boosted following Sequential Infection with Seasonal and Pandemic H1N1 Influenza Virus in Mice. J Virol. 2012;86(19):10302–7. doi: 10.1128/JVI.01336-12 . 22787225 10.1128/JVI.01336-12 PMC3457330

[39] Sliepen K, van Montfort T, Melchers M, Isik G, Sanders RW. Immunosilencing a highly immunogenic protein trimerization domain. J Biol Chem. 2015;290(12):7436–42. Epub 2015/01/31. doi: 10.1074/jbc.M114.620534 . 25635058 10.1074/jbc.M114.620534 PMC4367253

[40] Banzhoff A, Gasparini R, Laghi-Pasini F, Staniscia T, Durando P, Montomoli E, et al MF59-adjuvanted H5N1 vaccine induces immunologic memory and heterotypic antibody responses in non-elderly and elderly adults. PloS one. 2009;4(2):e4384 Epub 2009/02/07. doi: 10.1371/journal.pone.0004384 . 19197383 10.1371/journal.pone.0004384 PMC2634740

[41] Mulligan MJ, Bernstein DI, Winokur P, Rupp R, Anderson E, Rouphael N, et al Serological responses to an avian influenza A/H7N9 vaccine mixed at the point-of-use with MF59 adjuvant: a randomized clinical trial. JAMA. 2014;312(14):1409–19. doi: 10.1001/jama.2014.12854 . 25291577 10.1001/jama.2014.12854

[42] Pica N, Langlois RA, Krammer F, Margine I, Palese P. NS1-truncated live attenuated virus vaccine provides robust protection to aged mice from viral challenge. Journal of virology. 2012;86(19):10293–301. doi: 10.1128/JVI.01131-12 . 22787224 10.1128/JVI.01131-12 PMC3457311

[43] Henn AD, Wu S, Qiu X, Ruda M, Stover M, Yang H, et al High-resolution temporal response patterns to influenza vaccine reveal a distinct human plasma cell gene signature. Sci Rep. 2013;3:2327 doi: 10.1038/srep02327 . 23900141 10.1038/srep02327 PMC3728595

[44] Krammer F, Margine I, Hai R, Flood A, Hirsh A, Tsvetnitsky V, et al H3 stalk-based chimeric hemagglutinin influenza virus constructs protect mice from H7N9 challenge. J Virol. 2014;88(4):2340–3. doi: 10.1128/JVI.03183-13 . 24307585 10.1128/JVI.03183-13 PMC3911549

[45] Cobey S, Hensley SE. Immune history and influenza virus susceptibility. Curr Opin Virol. 2017;22:105–11. doi: 10.1016/j.coviro.2016.12.004 . 28088686 10.1016/j.coviro.2016.12.004 PMC5467731

[46] Park MS, Kim JI, Park S, Lee I, Park MS. Original Antigenic Sin Response to RNA Viruses and Antiviral Immunity. Immune Netw. 2016;16(5):261–70. doi: 10.4110/in.2016.16.5.261 . 27799871 10.4110/in.2016.16.5.261 PMC5086450

[47] Linderman SL, Hensley SE. Antibodies with ‘Original Antigenic Sin’ Properties Are Valuable Components of Secondary Immune Responses to Influenza Viruses. PLoS Pathog. 2016;12(8):e1005806 Epub 2016/08/19. doi: 10.1371/journal.ppat.1005806 . 27537358 10.1371/journal.ppat.1005806 PMC4990287

[48] Cortina-Ceballos B, Godoy-Lozano EE, Tellez-Sosa J, Ovilla-Munoz M, Samano-Sanchez H, Aguilar-Salgado A, et al Longitudinal analysis of the peripheral B cell repertoire reveals unique effects of immunization with a new influenza virus strain. Genome Med. 2015;7:124 doi: 10.1186/s13073-015-0239-y . 26608341 10.1186/s13073-015-0239-y PMC4658769

[49] King C. New insights into the differentiation and function of T follicular helper cells. Nat Rev Immunol. 2009;9(11):757–66. doi: 10.1038/nri2644 . 19855402 10.1038/nri2644

[50] Mastelic Gavillet B, Eberhardt CS, Auderset F, Castellino F, Seubert A, Tregoning JS, et al MF59 Mediates Its B Cell Adjuvanticity by Promoting T Follicular Helper Cells and Thus Germinal Center Responses in Adult and Early Life. J Immunol. 2015;194(10):4836–45. doi: 10.4049/jimmunol.1402071 . 25870238 10.4049/jimmunol.1402071

[51] Ansaldi F, Bacilieri S, Durando P, Sticchi L, Valle L, Montomoli E, et al Cross-protection by MF59-adjuvanted influenza vaccine: neutralizing and haemagglutination-inhibiting antibody activity against A(H3N2) drifted influenza viruses. Vaccine. 2008;26(12):1525–9. doi: 10.1016/j.vaccine.2008.01.019 . 18294741 10.1016/j.vaccine.2008.01.019

[52] Faenzi E, Zedda L, Bardelli M, Spensieri F, Borgogni E, Volpini G, et al One dose of an MF59-adjuvanted pandemic A/H1N1 vaccine recruits pre-existing immune memory and induces the rapid rise of neutralizing antibodies. Vaccine. 2012;30(27):4086–94. Epub 2012/04/24. doi: 10.1016/j.vaccine.2012.04.020 . 22521851 10.1016/j.vaccine.2012.04.020

[53] Heaton NS, Leyva-Grado VH, Tan GS, Eggink D, Hai R, Palese P. In vivo bioluminescent imaging of influenza a virus infection and characterization of novel cross-protective monoclonal antibodies. Journal of virology. 2013;87(15):8272–81. doi: 10.1128/JVI.00969-13 . 23698304 10.1128/JVI.00969-13 PMC3719835

[54] O’Hagan DT, Rappuoli R, De Gregorio E, Tsai T, Del Giudice G. MF59 adjuvant: the best insurance against influenza strain diversity. Expert review of vaccines. 2011;10(4):447–62. doi: 10.1586/erv.11.23 . 21506643 10.1586/erv.11.23

[55] Stephenson I, Nicholson KG, Hoschler K, Zambon MC, Hancock K, DeVos J, et al Antigenically distinct MF59-adjuvanted vaccine to boost immunity to H5N1. N Engl J Med. 2008;359(15):1631–3. doi: 10.1056/NEJMc0805274 . 18843132 10.1056/NEJMc0805274

[56] Khurana S, Coyle EM, Dimitrova M, Castellino F, Nicholson K, Del Giudice G, et al Heterologous prime-boost vaccination with MF59-adjuvanted H5 vaccines promotes antibody affinity maturation towards the hemagglutinin HA1 domain and broad H5N1 cross-clade neutralization. PloS one. 2014;9(4):e95496 doi: 10.1371/journal.pone.0095496 . 24755693 10.1371/journal.pone.0095496 PMC3995799

