# Supplementary material for: Broad cross-reactive IgG responses elicited by adjuvanted vaccination with recombinant influenza hemagglutinin (rHA) in ferrets and mice
Source: PLoS One. 2018 Apr 11;13(4):e0193680. doi: 10.1371/journal.pone.0193680 (PMC5894995; doi:10.1371/journal.pone.0193680)
Supplement: S1 File — Methods and results of single circle fluorescent forming unit based microneutralization assays. (S1 Table). (DOCX) [file pone.0193680.s001.docx]

**S1 File:**

**Single circle fluorescent forming units (FFU) based neutralization assay**

**Material and Methods**: A/HK68, A/Cal09, A/Puerto Rico/8/1934 (A/PR8) and B/Bris08 influenza viruses were provide by Dr. Martinez-Sobrido’s (University of Rochester,NY). A/Victoria/361/2011 influenza virus was obtained from the Influenza Research Resource (IRR, Manassas, VA). Viruses were titrated as previously described [[29](#_ENREF_29)]. Briefly, confluent plates of Madin-Darby canine kidney (MDCK, ATCC CCL-34) cells (96-well format, 5x10^4^ cells/well) were infected with 10-fold serial dilutions of tissue culture supernatants. At 8 hours post infection (hpi), cells were fixed and permeabilized using 4% formaldehyde, 0.5% triton-X100 in PBS for 30 minutes at room temperature. Cells were then washed thrice with PBS and incubated in PBS/2.5% bovine serum albumin (BSA) for one hour at room temperature. Cells were washed three more with PBS, and incubated with the IAV nucleoprotein (NP) monoclonal antibody (mAb) HB-65 (ATCC, H16-L10-4R5), diluted in 1% BSA for two hours at 37°C. After washing three more times with PBS, cells were incubated with a fluorescein isothiocyanate (FITC)-conjugated rabbit anti-mouse IgG secondary antibody (Dako) diluted 1:1,000 in 1% BSA in PBS, for one hour at 37°C. IAV NP-positive cells were visualized and enumerated to determine virus titers (fluorescent forming units, FFU/mL) using a fluorescence microscope. For microneutralization (MN) assays, two-fold serial dilutions of mouse sera were mixed with approximately 100 FFU of each virus and was left at room temperature for 60 minutes. Confluent MDCK cell monolayers were then inoculated with the serum/virus mixtures. After an absorption period of 60 minutes at room temperature, serum/virus mixtures were removed and replaced with DMEM (Gibco, Waltham, MA) supplemented with 0.3% BSA, 10 μg/mL gentamycin, and 1 μg/mL TPCK-trypsin. After incubation for 8 hpi at 37°C in air enriched with 5% CO_2_, infected cells were fixed and permeabilized with 0.5% Triton X-100 and 4% formaldehyde in PBS, and an immunofluorescence using an antibody specific for the viral NP protein was performed, as described above. The HA-specific neutralizing antibody titer was measured as the highest dilution of serum at which more than 90% of FFUs were inhibited.

**Results**

We assessed if the broadly reactive anti-rHA IgG induced by Addavax rHA vaccination correlated with higher levels of neutralizing antibodies capable of blocking viral infection. We measured the viral neutralization activity of mouse antisera generated from Addavax adjuvanted rHA vaccination against both vaccine specific and heterologous influenza viruses *in vitro* using a fluorescent forming units (FFU) based microneutralization assay[[29](#_ENREF_29)]. We found that the mouse sera generated by multiple Addavax adjuvanted vaccinations with A/HK68 rHA protein effectively neutralized homologous A/HK68 virus, and it also cross-neutralized the infection of A/Vic11 virus *in vitro* (S1 Table). Although A/Vic11 is an H3 influenza virus, within the same subtype as A/HK68, A/Vic11 exhibits significant antigenic drift as compared with A/HK68 (S3 Fig). These results correlate with our above observation showing that adjuvanted rHA vaccination induces broader cross-reactivity than infection. This finding is also consistent with clinical studies showing that Addavax adjuvanted influenza vaccination induces broadly reactive neutralizing antibodies in human subjects [[25](#_ENREF_25), [43](#_ENREF_43)]. But we could not detect the neutralization activity against the A/PR8 (H1N1) virus in the antisera of A/Cal09 vaccination. Interestingly, we observed that anti-HA antibodies from sera generated by adjuvanted A/Cal09 rHA vaccination reproducibly showed higher titers (2-fold) against the heterologous A/Vic11 virus (H3) and against the homologous A/Cal09 (4-fold) than the infection strategy. We repeated and used A/Per09, other H3 influenza virus, and observed the similar results.
