# Supplementary material for: Broad cross-reactive IgG responses elicited by adjuvanted vaccination with recombinant influenza hemagglutinin (rHA) in ferrets and mice
Source: PLoS One. 2018 Apr 11;13(4):e0193680. doi: 10.1371/journal.pone.0193680 (PMC5894995; doi:10.1371/journal.pone.0193680)
Supplement: S1 Table — Neutrilzation assays were performed against against A/HK68, A/Cal09, A/Puerto Rico/8/1934 (A/PR8) and B/Bris08 influenza strains. Sera from the following groups were compared: Addavax Control, A/Cal09 Infection, rHA A/Cal09 + Addavax, A/HK68 Infection, and rHA A/HK68 + Addavax. Results demonstrated significantly higher titers that neutralized homologous and cross-strain influenza virus. (DOCX) [file pone.0193680.s002.docx]

**S1 Table. Serum neutralization titers induced after viral infection or Addavax Adjuvanted**

**rHA vaccination with A/Cal09(H1) or A/HK68 (H3) influenza virus strains***

| **Groups** | **Tested influenza viruses** | | | | |
| --- | --- | --- | --- | --- | --- |
|  | A/Cal09 (H1) | A/PR8 (H1) | A/HK68 (H3) | A/Per09 (H3) | B/Bris08 (B) |
| Addavax Control | ≤ 20 | ≤ 20 | ≤ 20 | 40 | ≤ 20 |
| A/Cal09 Infection | 640 | ≤ 20 | ≤ 20 | 40 | ≤ 20 |
| rHA A/Cal09 + Addavax | 1280 | ≤ 20 | ≤ 20 | 80 | ≤ 20 |
| A/HK68 Infection | ≤ 20 | ≤ 20 | 640 | 160 | ≤ 20 |
| rHA A/HK68 + Addavax | ≤ 20 | ≤ 20 | 1280 | 160 | ≤ 20 |

* experiments were repeated twice, in duplicates.
