# Supplementary material for: Broad cross-reactive IgG responses elicited by adjuvanted vaccination with recombinant influenza hemagglutinin (rHA) in ferrets and mice
Source: PLoS One. 2018 Apr 11;13(4):e0193680. doi: 10.1371/journal.pone.0193680 (PMC5894995; doi:10.1371/journal.pone.0193680)

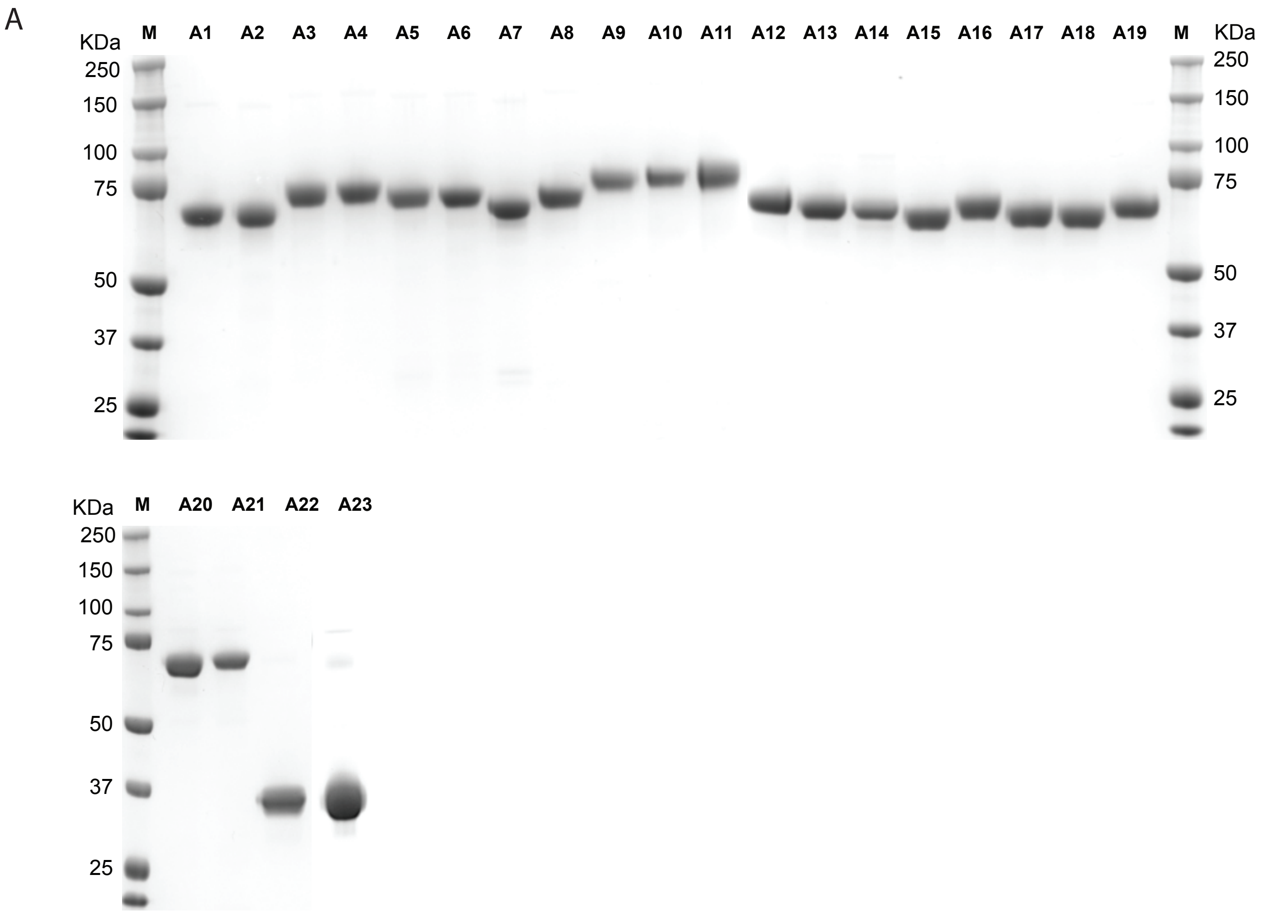

No.	HA strains	Abbreviation
**A1**	A/South Carolina/1/1918 (H1)	A/SC18
**A2**	A/PR/8/1934 (H1)	A/PR8
**A3**	A/USSR/1977 (H1)	A/USSR77
**A4**	A/Texas/36/1991 (H1)	A/Tex91
**A5**	A/New Caledonia/20/1999 (H1)	A/NewCal99
**A6**	A/California/04/2009 (H1)	A/Cal09
**A7**	A/Japan/305/1957 (H2)	A/Jap57
**A8**	A/Hong Kong/1/1968 (H3)	A/HK68
**A9**	A/Wisconsin/67/2005 (H3)	A/Wis05
**A10**	A/Perth/16/2009 (H3)	A/Per09
**A11**	A/Victoria/361/2011 (H3)	A/Vic11
**A12**	A/Vietnam/1204/2004 (H5)	A/Vie04
**A13**	A/Indonesia/05/2005 (H5)	A/Ind05
**A14**	A/Taiwan/2/13 (H6)	A/TW13
**A15**	A/mallard/Netherlands/12/2000 (H7)	A/Net00
**A16**	A/rhea/North Carolina/39482/93 (H7)	A/NC93
**A17**	A/Shanghai/1/2013 (H7)	A/SH13
**A18**	A/Anhui/1/2013 (H7)	A/AH13
**A19**	A/guinea fowl/HongKong/WF10/1999 (H9)	A/gfHK99
**A20**	A/Vie04 head and A/PR34 stalk	cH5/1PR
**A21**	A/Vie04 head and A/Cal09 stalk	cH5/1Cal
**A22**	H5 head (Ind05)	H5 head
**A23**	H9 head (HK99)	H9 head

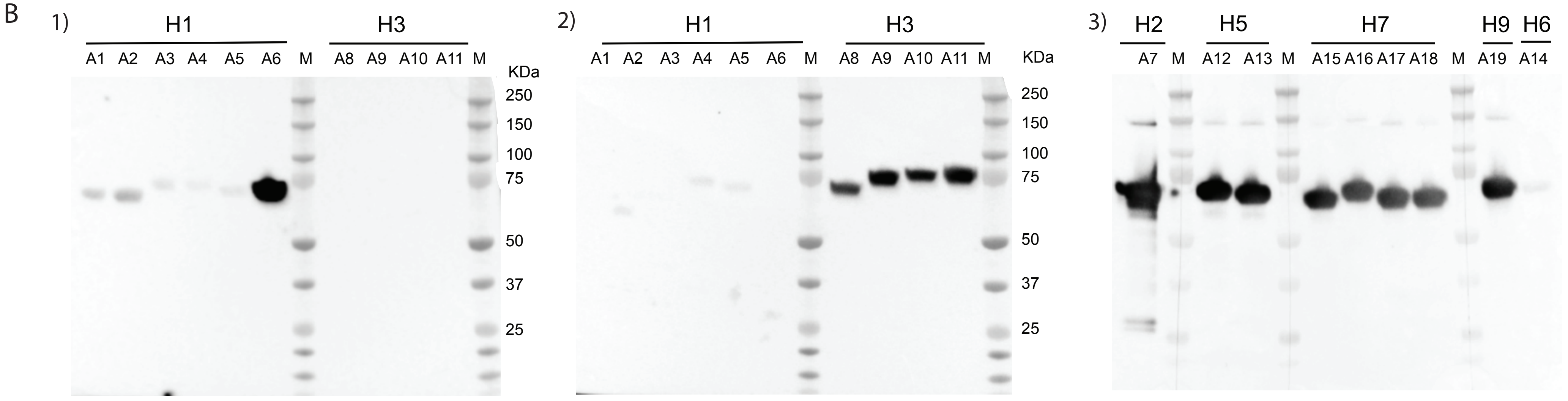

Supplement: S1 Fig — The ectodomain of HA0 genes of influenza viruses were expressed in the baculovirus system using a modified pFastBac vector (Invitrogen) that harbors a hexahistidine tag at the C-terminal. The rHAs were purified with Ni-NTA resin (Qiagen) and concentrated with Amicon Ultra 30K filters (Millipore). A. The SDS-PAGE gel of rHAs preparations. The purified rHAs were separated and analyzed by 4–12% Bis-Tris NuPage gels (Invitrogen). B. The antigenic characteristics of rHAs of influenza virus were verified by the e Western-blot analysis of rHAs preps. Purified recombinant HAs were separated on SDS-PAGE and transferred to nitrocellulose membrane (0.45μm, Bio-Rad). After the membrane was blocked with 5% milk in TBST buffer (500 mM NaCl, 20 mM pH 7.5 Tris–HCl, 0.05% Tween-20)) for 30min at room temperature, it was detected with ferret polyclonal antisera of influenza A subtype viruses provided by Influenza Reagent Resource (IRR) 1) antiserum to A/California/04/2009(H1N1) (FR-359); 2) antiserum to A/Perth/16/2009 (H3N2) (FR-446); 3) antiserum to A/Japan/305/1957 (H2N2) (FR-891), A/Vietnam/1203/2004 (H5N1) (FR-708), A/Netherlands/219/2003 (H7N7) (FR-890) and A/HongKong/1073/1999 (H9N2) (FR-889). (PDF) [file pone.0193680.s003.pdf]
