# Supplementary figures and images for: Broad cross-reactive IgG responses elicited by adjuvanted vaccination with recombinant influenza hemagglutinin (rHA) in ferrets and mice
Source: PLoS One. 2018 Apr 11;13(4):e0193680. doi: 10.1371/journal.pone.0193680 (PMC5894995; doi:10.1371/journal.pone.0193680)

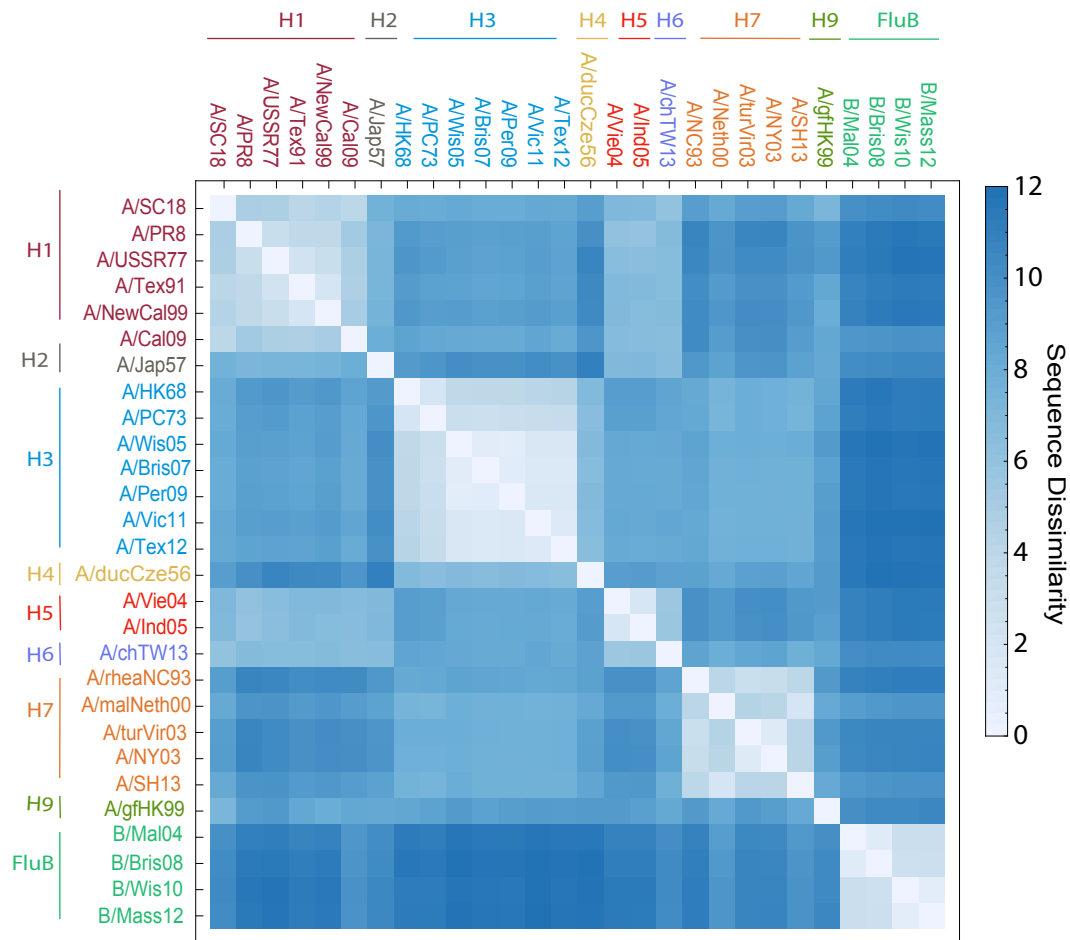

Supplement: S3 Fig — Sequence dissimilarity of HA molecular based on the protein sequences of rHAs using Euclidean distance measurement and protein feature vector method were performed to estimate the distance between the actual and theoretical sequence based on the binomial and uniform distributions [34, 35], and metric multidimensional scaling was performed using custom Mathematica code. (PDF) [file pone.0193680.s005.pdf]
